# Glucose 6-phosphate dehydrogenase 6-phosphogluconolactonase: characterization of the *Plasmodium vivax* enzyme and inhibitor studies

**DOI:** 10.1186/s12936-019-2651-z

**Published:** 2019-01-25

**Authors:** Kristina Haeussler, Isabell Berneburg, Esther Jortzik, Julia Hahn, Mahsa Rahbari, Norma Schulz, Janina Preuss, Viktor A. Zapol’skii, Lars Bode, Anthony B. Pinkerton, Dieter E. Kaufmann, Stefan Rahlfs, Katja Becker

**Affiliations:** 1 Biochemistry and Molecular Biology, Interdisciplinary Research Center, Justus Liebig University, Heinrich-Buff-Ring 26-32, 35392 Giessen, Germany; 2 Conrad Prebys Center for Chemical Genomics, Sanford Burnham Prebys Medical Discovery Institute, La Jolla, CA 92037 USA; 3 Department of Pediatrics, University of California San Diego, San Diego, CA 92093 USA; 4 Institute of Organic Chemistry, Clausthal University of Technology, 38678 Clausthal-Zellerfeld, Germany

**Keywords:** Glucose 6-phosphate dehydrogenase, Inhibitors, *Plasmodium vivax*, *Plasmodium falciparum*, SNP, Malaria, Drug target, Redox balance, Post-translational modification

## Abstract

**Background:**

Since malaria parasites highly depend on ribose 5-phosphate for DNA and RNA synthesis and on NADPH as a source of reducing equivalents, the pentose phosphate pathway (PPP) is considered an excellent anti-malarial drug target. In *Plasmodium*, a bifunctional enzyme named glucose 6-phosphate dehydrogenase 6-phosphogluconolactonase (GluPho) catalyzes the first two steps of the PPP. *Pf*GluPho has been shown to be essential for the growth of blood stage *Plasmodium falciparum* parasites.

**Methods:**

*Plasmodium vivax* glucose 6-phosphate dehydrogenase (*Pv*G6PD) was cloned, recombinantly produced in *Escherichia coli*, purified, and characterized via enzyme kinetics and inhibitor studies. The effects of post-translational cysteine modifications were assessed via western blotting and enzyme activity assays. Genetically encoded probes were employed to study the effects of G6PD inhibitors on the cytosolic redox potential of *Plasmodium*.

**Results:**

Here the recombinant production and characterization of *Pv*G6PD, the C-terminal and NADPH-producing part of *Pv*GluPho, is described. A comparison with *Pf*G6PD (the NADPH-producing part of *Pf*GluPho) indicates that the *P. vivax* enzyme has higher K_*M*_ values for the substrate and cofactor. Like the *P. falciparum* enzyme, *Pv*G6PD is hardly affected by *S*-glutathionylation and moderately by *S*-nitrosation. Since there are several naturally occurring variants of *Pf*GluPho, the impact of these mutations on the kinetic properties of the enzyme was analysed. Notably, in contrast to many human G6PD variants, the mutations resulted in only minor changes in enzyme activity. Moreover, nanomolar IC_50_ values of several compounds were determined on *P. vivax* G6PD (including ellagic acid, flavellagic acid, and coruleoellagic acid), inhibitors that had been previously characterized on *Pf*GluPho. ML304, a recently developed *Pf*GluPho inhibitor, was verified to also be active on *Pv*G6PD. Using genetically encoded probes, ML304 was confirmed to disturb the cytosolic glutathione-dependent redox potential of *P. falciparum* blood stage parasites. Finally, a new series of novel small molecules with the potential to inhibit the *falciparum* and *vivax* enzymes were synthesized, resulting in two compounds with nanomolar activity.

**Conclusion:**

The characterization of *Pv*G6PD makes this enzyme accessible to further drug discovery activities. In contrast to naturally occurring G6PD variants in the human host that can alter the kinetic properties of the enzyme and thus the redox homeostasis of the cells, the naturally occurring *Pf*GluPho variants studied here are unlikely to have a major impact on the parasites’ redox homeostasis. Several classes of inhibitors have been successfully tested and are presently being followed up.

**Electronic supplementary material:**

The online version of this article (10.1186/s12936-019-2651-z) contains supplementary material, which is available to authorized users.

## Background

*Plasmodium falciparum* causes the most severe form of malaria and is responsible for nearly all malaria-related deaths [1, 2]. In contrast, *Plasmodium vivax* infections are usually not fatal; however, they can cause severe anaemia and can be followed by frequent relapses that can, for example, seriously affect the development of children [3]. Therefore, early detection and appropriate treatment are essential in containing the consequences of *P. vivax* infection [4]. Currently, the World Health Organization (WHO) recommends an artemisinin-based combination therapy (ACT) for the treatment of uncomplicated *falciparum* malaria, and chloroquine or ACT for the treatment of uncomplicated *vivax *malaria. In many cases, primaquine is used to eradicate liver stages and prevent relapses [2, 5]. However, spreading resistance of both *P. falciparum* and *P. vivax* to chloroquine underlines the importance of continuing drug development efforts [2]. Here, a major challenge is to maintain *P. vivax* in continuous in vitro culture [6].

In *Plasmodium*, the pentose phosphate pathway (PPP) is supposed to be the main source of ribulose 5-phosphate and NADPH, providing reducing equivalents for downstream glutathione and thioredoxin-dependent reactions, which are crucial for redox balance, redox regulatory, and redox signaling processes [7–9]. Redox metabolism represents an important drug target and has been shown to be involved in the mechanism of action of anti-malarial drugs as well as in drug resistance [8, 10–12]. As demonstrated for *P. falciparum* and other *Apicomplexa* [13, 14], the PPP of these parasites contains a bifunctional enzyme consisting of glucose 6-phosphate dehydrogenase (G6PD) and 6-phosphogluconolactonase (6PGL). This enzyme is referred to as GluPho. Recombinant *P. falciparum* GluPho has already been characterized in detail [14]. In 2015, attempted double crossover disruption indicated that *Pf*GluPho is essential for the growth of *P. falciparum* blood stages, confirming *Pf*GluPho as a drug target [15]. Therefore a high-throughput-compatible assay was developed, and various compound libraries were screened against the G6PD activity of *Pf*GluPho, identifying two nanomolar inhibitors (ML276 and ML304) that are selective for *P. falciparum* G6PD over hG6PD and inhibit the growth of *P. falciparum* 3D7 in vitro [16–18].

The redox system of *P. vivax* shows high homologies to *P. falciparum*, including the presence of a bifunctional GluPho in the PPP. However, apart from a 2-Cys, a 1-Cys peroxiredoxin [19, 20] and glutathione *S*-transferase [21], the biochemistry of proteins contributing to the *P. vivax* cellular redox network has so far hardly been characterized [22]. Attempts to transfer knowledge of the *P. falciparum* redox system and its inhibitors to *P. vivax* can, therefore, be very valuable in facilitating drug discovery against *vivax* malaria.

It is well known that exposure to *Plasmodium* parasites has left marks on the human genome. Several genetic disorders such as sickle cell disease, thalassaemia, and G6PD deficiency show the same geographic distribution as *falciparum* and *vivax* malaria and offer a certain degree of protection from the infection (for review see [23]). G6PD deficiency is found in around 400 million people, especially in malaria-endemic regions, and can be caused by over 200 known mutations in the hG6PD-encoding gene [24]. The mild and most common mutations result in reduced G6PD activity, and their protective effect against malaria is most likely based on enhanced phagocytosis of parasitized G6PD-deficient erythrocytes [25–27]. The *Plasmodium* genome also shows naturally occurring variations [28, 29] that help the parasites deal with evolutionary selection pressure, including host immunity and drug treatment [30, 31]. Sequencing the genome of *P. falciparum* derived from the blood of malaria-infected patients identified several mutations in the *Pf*GluPho gene (PlasmoDB). These mutations have so far not been studied with respect to their functional consequences for enzyme activity.

Here the biochemical and kinetic characterization of recombinant *P. vivax* G6PD, as well as of different variants of *P. falciparum* GluPho occurring in the field, are reported. Several known and newly synthesized small molecule compounds are further described, with strong inhibitory effects on *Pv*G6PD that might be used as a starting point for drug discovery against *vivax* malaria.

## Methods

### Drugs and chemicals

All chemicals used were of the highest available purity and were obtained from Roth (Karlsruhe, Germany), Sigma-Aldrich (Steinheim, Germany), or Merck (Darmstadt, Germany). NADP^+^ was purchased from Biomol (Hamburg, Germany), Ni–NTA agarose from Cube Biotech (Monheim am Rhein, Germany), RPMI 1640 medium from Gibco (Paisley, United Kingdom), and artemisinin (ATS) and chloroquine (CQ) from Sigma-Aldrich (Steinheim, Germany). ML304 was synthesized as described previously [18]. Ellagic acid (EA) was purchased from Sigma-Aldrich (Steinheim, Germany). Two synthetic derivatives of EA, flavellagic acid (FEA) and coruleoellagic acid (CEA), were synthesized as described previously [32–34]. WR99210 was kindly supplied by Jacobus Pharmaceuticals (New Jersey, USA). Stock solutions of diamide (DIA), DTT, and CQ were dissolved in sterile ddH_2_O, while all others were dissolved in DMSO.

### Cloning *Pv*GluPho and *Pv*G6PD

The gene of *Pv*GluPho (PlasmoDB accession number PVX_117790) is composed of one exon with 2784 bp and does not contain an EcoRI recognition site. The gene was amplified via PCR using a *vivax* EcoRI DNA-library-obtained by MR4—as a template using primers (OPvGPNatatGGATCCGATTGCCAGGCGCTGGCGAA and OPvGPCatatGTCGACTCAGTTGATGTCCAACAAGTCGT), introducing restriction sites for *BamHI* and *SalI* (underlined). The construct was cloned into the cloning vector pBluescriptSK+, and the insert was verified via in-house sequencing. For heterologous overexpression, the construct was subcloned into the vectors pQE30 and pET28a using the same restriction sites.

The G6PD part of *Pv*GluPho (*Pv*G6PD, aa 310-927) was amplified via PCR (bp 930–2784) using primer sense with a *BamHI* recognition site (underlined, 5′-CGGGATCCCTGAATAGGAGGGAATGCCTA-3′), and antisense with a *SalI* site (5′-atatGTCGACTCAGTTGATGTCCAACAAGTCGT-3′), and was cloned into the vector pQE30 for heterologous overexpression.

### Cloning and heterologous overexpression of the hGrx1–roGFPF2 construct

The sensor hGrx1–roGFP2 (human glutaredoxin 1-reduction–oxidation sensitive green fluorescent protein 2) was recently transiently expressed in 3D7 *P. falciparum* parasites by using the pARL1a(+) expression vector. Heterologous overexpression of hGrx1–roGFP2 for the evaluation of the in vitro interactions of ML304 and anti-malarial drugs with the recombinant redox probe was performed according to [35].

### Site-directed mutagenesis of *Pf*GluPho

Multi-sequence alignments of G6PDs from various species showed the presence of a prominent SS pair at the C-terminal end of GluPho from *P. falciparum* and other plasmodial species. This is absent in other G6PDs such as man and *Saccharomyces cerevisiae* (Additional file 1). Amongst other residues, these two serines could be phosphorylated as demonstrated by several groups [36–38]. Therefore, they were mutated to glutamate in order to create phosphomimetic mutants (*Pf*GluPho^S899E^, *Pf*GluPho^S900E^) [39]. This allowed studying possible consequences of protein phosphorylation on the activity and substrate affinity of *Pf*GluPho. Moreover, naturally occurring SNPs of *Pf*GluPho were chosen based on data provided in PlasmoDB. To analyse the influence of these SNPs on *Pf*GluPho activity, different mutations were introduced into *Pf*GluPho wt via site-directed mutagenesis. Single mutations were introduced at Ser315 (*Pf*GluPho^S315Y^), Leu395 (*Pf*GluPho^L395F^), and Phe507 (*Pf*GluPho^F507L^). The coding sequence for *Pf*GluPho wt (PlasmoDB PF3D7_1453800) was cloned and expressed as described elsewhere [14]. To obtain the mutants, *Pf*GluPho wt was used as a template, *Pfu* polymerase (Promega, Mannheim, Germany), and the mutagenesis primers listed in Additional file 2. Methylated, non-mutated template plasmids were digested with *Dpn*I (Thermo Scientific, Dreieich, Germany), and the correct mutations were confirmed via in-house sequencing.

### Heterologous overexpression and purification of recombinant proteins

*Pf*GluPho wt, its mutants, *Pf*G6PD, and the human homologue hG6PD were produced as described previously [14]. For the heterologous overexpression of *Pv*GluPho, numerous *Escherichia coli* strains were tested under various conditions. However, none of the conditions tested so far yielded active proteins. Therefore, *Pv*G6PD was heterologously overexpressed in *E. coli* M15 (Qiagen, Hilden, Germany) containing the plasmid pRAREII (Novagen, Darmstadt, Germany) for overcoming the codon bias in *E. coli*. Cells were grown in terrific broth medium, supplemented with carbenicillin, kanamycin (both 50 µg mL^−1^), and chloramphenicol (12.5 µg mL^−1^) at 37 °C. After an optical density at 600 nm of 0.6–0.8 was reached, overexpression was induced with 1 mM isopropyl-β-d-thiogalactopyranoside (IPTG). After 20 h shaking at room temperature, cells were harvested via centrifugation (15 min, 12,000 *g*, 4 °C), resuspended in 500 mM NaCl, 50 mM Tris, pH 7.8, and mixed with protease inhibitors (100 µM phenylmethylsulfonyl fluoride, 150 nM pepstatin A, 40 nM cystatin). The cells were lysed with lysozyme and DNaseI for at least 14 h at 4 °C, sonicated (three cycles of 30 s at 70% and 4 °C), and centrifuged (30 min, 25,000 *g*, 4 °C). The supernatant was applied to a Ni–NTA agarose column; unspecifically bound proteins were eluted with 500 mM NaCl, 50 mM Tris, pH 7.8, and increasing imidazole concentrations from 10 to 40 mM imidazole. The recombinant proteins were eluted under the same buffer conditions containing 80–500 mM imidazole. To improve the purity of the proteins, size exclusion chromatography was performed as described in [14], followed by a control of the final purity of protein via SDS-PAGE using a 12% gel. The protein was stored with 50% glycerol at −80 °C.

### Enzymatic characterization of *Pf*GluPho variants and *Pv*G6PD

The G6PD activity of *Pf*GluPho variants was measured spectrophotometrically as described for the wt enzyme [14]. Kinetic characterization of *Pv*G6PD was performed using the same assay system at an Evolution 300 spectrophotometer (Thermo Scientific, Dreieich, Germany). In brief, the initial reduction of NADP^+^ to NADPH by *Pv*G6PD was monitored by measuring the absorbance at 340 nm. The 500 µL reaction mixture contained 200 µM NADP^+^ and varying concentrations of *Pv*G6PD in an assay buffer of 100 mM Tris–HCl, pH 8.0, 10 mM MgCl_2_, and 0.5 mM EDTA at 25 °C. The reaction was started by adding 800 µM G6P. The *K*_M_ for NADP^+^ was determined by titrating NADP^+^ from 2 to 400 µM at 800 µM G6P; the *K*_M_ for G6P was measured accordingly by titrating G6P from 2 to 1200 µM at 200 µM NADP^+^. *K*_M_ values were calculated using GraphPad Prism. Since the *K*_M_ for G6P was considerably higher than the value measured for *Pf*GluPho [14], a concentration of 800 µM G6P was defined as substrate saturation for *Pv*G6PD.

### Protein S-glutathionylation of *Pv*G6PD, *Pf*G6PD, and *Pf*GluPho

To investigate the susceptibility of *Pv*G6PD, *Pf*G6PD, and *Pf*GluPho to *S*-glutathionylation, purified enzymes were reduced with 5 mM DTT for 30 min at 4 °C in 500 mM NaCl, 50 mM Tris, pH 7.8. Afterwards, DTT was removed using Zeba™ spin desalting columns (ThermoFisher Scientific, Waltham, MA, USA) according to the instructions of the manufacturer. The proteins were adjusted to a final concentration between 18.5 and 30.3 µM and incubated with 0, 2, 6, or 10 mM GSSG for 10 min at 37 °C, followed by desalting as described above. Afterwards, the samples were applied to non-reducing SDS-PAGE, while reducing SDS-PAGE was used to test the reversibility of the potential modification. As a positive control, recombinant *Pf*Prx1a was used, which had been previously proved to be part of the glutathionylome of *P. falciparum* [40]. Ponceau staining of the membrane after blotting served as a loading control. *S*-glutathionylated protein was detected via semi-dry western blotting using a monoclonal anti-glutathione antibody (Virogen, Massachusetts, USA; diluted 1:250 in 5% nonfat milk with Tris-buffered saline Tween-20 (TBST)) and a horseradish peroxidase-conjugated goat anti-mouse antibody (Dianova, Hamburg, Germany; diluted 1:10,000 in 5% nonfat milk with TBST). Experiments were carried out in at least duplicate using different batches of enzyme.

### Protein *S*-nitrosation of *Pv*G6PD, *Pf*G6PD, and *Pf*GluPho

To examine the susceptibility of *P. vivax* G6PD and *P. falciparum* G6PD/GluPho to *S*-nitrosation, a biotin switch assay was performed as described in [41, 42], finally detecting *S*-nitrosated thiol groups via α-biotin western blot analysis. In brief, pre-reduced proteins were adjusted to a concentration between 7.4 and 12.1 µM and incubated with 0 or 1 mM *S*-nitroso-glutathione (GSNO) in 50 mM Tris, pH 7.4, 1 mM EDTA, and 0.2 mM neocuproine (GSNO buffer) for 1 h at 22 °C in the dark. The reaction was stopped by adding 100% ice-cold acetone, followed by protein precipitation for 30 min at −20 °C. The samples were centrifuged (8000 *g*, 4 °C, 5 min); the supernatant was discarded; and the pellets were washed three times with 70% ice-cold acetone, each time followed by centrifugation (first centrifugation 8000 *g*, followed by 5000 *g*, 4 °C, 5 min). Residual cysteine thiols were blocked by resuspending the pellets in 8 M urea, 50 mM Tris, pH 8.0, 1 mM EDTA, 0.1 mM neocuproine (blocking buffer) containing 200 mM iodoacetamide (IAA) and incubation in the dark for 45 min at 50 °C. The reaction was stopped by adding 100% ice-cold acetone, protein precipitation, and washing the pellets as described above. Afterwards, the pellets were dissolved in 4 M urea, 50 mM Tris, pH 8.0, 1 mM EDTA, 0.01 mM neocuproine (labelling buffer) containing 20 mM NaAsc and 0.2 mM iodoacetyl-PEG2-biotin and incubated for 1 h in the dark at 25 °C. Samples without NaAsc served as negative controls. The reaction was stopped as described above. After washing, the pellets were resuspended in blocking buffer and applied to SDS-PAGE. *S*–nitrosated protein was detected indirectly via semi-dry western blotting using a monoclonal anti-biotin antibody (Santa Cruz, Dallas, USA; Biotin Antibody (33): sc-101339; diluted 1:1000 in 5% nonfat milk with TBST) and a horseradish peroxidase-conjugated goat anti-mouse antibody (Dianova, Hamburg, Germany; diluted 1:10,000 in 5% nonfat milk with TBST). As a loading control, SDS-PAGE was performed, followed by Coomassie staining the proteins using 18 µL (1:10 dilution) of the samples used for western blotting (2 µL were applied to SDS-PAGE for western blotting, 1:10 dilution for *Pf*GluPho, and 1:50 dilution for *Pf*G6PD and *Pv*G6PD due to higher protein concentrations).

To study the impact of GSNO-mediated *S*-nitrosation on the activity of the enzymes, 1.9–3.0 µM of reduced enzyme was incubated with 0–1000 µM GSNO in GSNO buffer for up to 180 min at 22 °C in the dark. Neocuproine was added as a chelating agent in order to decrease S-NO breakage by copper ions, which had been previously observed [43]. Aliquots were taken at different time points to measure the activity, using the assay described above. To study the reversibility of *S*-nitrosation, *Pv*G6PD, *Pf*G6PD, and *Pf*GluPho were nitrosated as described above, followed by incubation with 5 mM DTT for 30 min at 4 °C. Afterwards, the enzyme activity was determined using the assay described above.

### Chemical synthesis of novel potential *Pf*GluPho inhibitors

Based on an initial screening approach, a set of compounds with the potential to inhibit *Pf*GluPho has been synthesized. The structures of the most active synthetic compounds are depicted in Fig. 8. The synthesis and characterization of pyrimidine **2** (vz1731), pyridine **4** (vz1732), ketene aminal **8** (vz0882), carboxylic acid **10** (vz1204), aldehyde **13** (vz0288), and imidazolidine **18** (vz0909) are described in Additional file 3; the synthesis and characterization of imidazolidines **19** (vz0914) and **20** (vz0005) are described in [44] and [45], respectively, and thiophene **21** (vz0527) in [46]. All spectral data was in accordance with the literature.

### Enzymatic characterization of compounds

To identify potential inhibitors of *Pv*G6PD, compounds with already known effects on *Pf*GluPho (ellagic acid and derivatives, ML304) were tested. Additionally, several compound libraries of the Helmholtz Center for Infection Research, Brunswick, were screened against *Pf*GluPho using the high-throughput-compatible assay systems described in Preuss et al. [16, 17]. The compound library synthesized by DK and VZ mainly consisted of structurally diverse heterocyclic compounds that had already proved to be microbiologically active in other areas such as anti-infectants and insecticides for plant protection. Based on structure-activity relationship studies, a set of new potential G6PD inhibitors (Fig. 8) was then synthesized and assessed. First, the IC_50_ values of the compounds were determined, followed by mechanistic characterization of the mode of action. To test for selectivity of the compounds, the IC_50_ values of the compounds on *Pv*G6PD, *Pf*GluPho, and hG6PD were determined in parallel. Compounds **8** (vz0882), **18** (vz0909), and **19** (vz0914) were less stable in the assay buffer; the compounds with the highest efficiency, **21** (vz0527), **2** (vz1731), and **4** (vz1732), were only active when the powder was freshly dissolved.

All measurements were performed in 96-well plates, half area (Greiner Bio-One, Frickenhausen, Germany) using a Tecan Infinite M200 plate reader (Maennedorf, Switzerland). The compounds were dissolved in 100% DMSO and afterwards diluted in assay buffer to the required concentrations. Different compound concentrations were added to a mixture of enzyme and NADP^+^ in assay buffer. Concentrations of substrates differed depending on the enzyme; an overview is given in the Additional file 4. The reaction was started by adding G6P, and the increase of NADPH was monitored by measuring the absorbance at 340 nm. To increase the sensitivity of the assay, some measurements were reproduced, detecting the fluorescence of NADPH at ex340/em460. The assay contained both substrates either in saturation or at concentrations close to *K*_M_ to facilitate the identification of competitive inhibitors. A negative control containing no compound was defined as 0% inhibition; a positive control lacking the substrate G6P was defined as 100% inhibition. For evaluations using GraphPad Prism and Microsoft Excel, only a linear increase in absorbance/fluorescence was considered. For the mechanistic characterization of the compounds, either NADP^+^ or G6P were titrated at different compound concentrations around the IC_50_, while keeping the second substrate in saturation. The initial slope was plotted as Michaelis–Menten curves and Lineweaver–Burk plots using GraphPad Prism; *K*_M_ values and V_max_ were calculated with GraphPad Prism software.

### Cell culture and transfection of *P. falciparum*

The CQ-sensitive *P. falciparum* 3D7 and NF54-*attB* strains were cultured as described in [47, 48], respectively. Briefly, the strains were propagated in red blood cells (RBCs) (A+) in RPMI 1640 medium supplemented with 0.5% w/v Albumax, 9 mM glucose, 0.2 mM hypoxanthine, 2.1 mM l-glutamine, 25 mM HEPES, and 22 µg mL^−1^ gentamycin at 3.3% haematocrit and 37 °C in a gaseous mixture consisting of 3% O_2_, 3% CO_2_, and 94% N_2_. *Plasmodium falciparum* parasites were synchronized with 5% (w/v) sorbitol [49]. *Plasmodium falciparum* trophozoites were enriched via magnetic separation [50]. Cell lysates were obtained via saponin lysis [51]. Parasitaemia was counted on Giemsa-stained blood smears. *Plasmodium falciparum* was transfected as described in [35].

### In vitro characterization of ML304 using recombinant hGrx1-roGFP2

ML304 was used at concentrations of 10 nM to 1 mM in degassed standard reaction buffer (100 mM potassium phosphate, 1 mM EDTA, pH 7.0). The in vitro interaction of the pharmacologically used anti-malarial drugs ATS and CQ with the purified hGrx1–roGFP2 protein had been determined previously and did not have an effect at the concentrations used in this study [35]. The characterization of the in vitro interaction of hGrx1–roGFP2 with ML304 was performed according to Schuh et al. [52].

### Confocal live cell imaging and image processing

A Leica confocal system TCS SP5 inverted microscope equipped with the objective HCX PL APO 63.0 × 1.30 GLYC 37 °C UV connected to a 37 °C temperature chamber was used. The argon laser power was set to 20%; scanning was performed at 400 Hz frequency and at a 512 × 512 pixel resolution. The smart gain and smart offset were 950 V and −0.9%, respectively. With a sequential scan, the hGrx1–roGFP2 probe was excited at 405 nm and at 488 nm and emission was detected at 500–550 nm. Laser intensity was adjusted to match the full dynamic range of the probe to the dynamic range of the detector (405 nm: 10%, 488 nm: 4%). Autofluorescence images were simultaneously taken at ex 405/em430–450 and individually defined together with the background for every image, but no fluorescence signal could be detected. The Leica LAS AF Lite software for fluorescence analysis was used. The 405/488 nm ratio was calculated. The graphs were plotted using the GraphPad Prism 5 software (San Diego, CA, USA). For live cell imaging, only parasites showing fluorescent signals at both 405 and 488 nm excitation and an intact host cell were chosen.

### Live cell imaging with a fluorescence plate reader

NF54^[hGrx1–roGFP2]^ parasites were washed and resuspended after incubation in pre-warmed Ringer’s solution to a concentration of 2.0 × 10^5^ iRBCs/µL. 10 µL of cells (1 × 10^6^ iRBCs) were transferred to each well of a 384-well plate (black, flat bottom, Greiner Bio-One, Frickenhausen, Germany) and were measured with the plate reader Clariostar (BMG Labtech, Ortenberg, Germany) with excitation wavelengths at 405 nm and 475 nm (emission 510 nm). The gain of the 405 nm and 475 nm excitation wavelengths was adjusted to match the full dynamic range of the hGrx1–roGFP2 redox sensor. The 405/475 nm ratio was then calculated. The graphs were plotted using the GraphPad Prism 5 software (San Diego, CA, USA). A one-way ANOVA test with 95% confidence intervals with the Dunnett’s multiple comparison test (GraphPad Prism 5.0) was applied for statistical analysis of significance (*p < 0.05; **p < 0.01; ***p < 0.001).

### Effects of anti-malarial compounds on redox homeostasis

The effect of ML304 and the anti-malarial drugs ATS and CQ as reference drugs on *P. falciparum* were investigated in 4 h and 24 h incubation experiments. The half-maximal effective concentration (EC_50_) of ML304 on *P. falciparum* 3D7 (471.3 ± 39.5 nM) asexual blood stages was determined with the [^3^H]-incorporation assay and also used as a reference value for setting up the NF54^[hGrx1–roGFP2]^ experiments. The EC_50_ of ATS (4.4 nM) and CQ (8.6 nM) were determined previously [35]. For 4 h experiments, trophozoite stage parasites (26–30 h) of 3D7^[hGrx1–roGFP2]^ and NF54^[hGrx1–roGFP2]^ (6–8% parasitaemia) were magnetically enriched (Miltenyi Biotec, Germany), counted by using a Neubauer hemocytometer (Brand GmbH, Germany), and returned to cell culture (at 2.0 × 10^4^ trophozoites μL^−1^) for at least 1 h to recover. 1.0 × 10^6^ cells in 100 μL cell culture medium were placed into LoBind tubes (Eppendorf, Hamburg, Germany) for 4 h incubation experiments. The parasites were treated with ML304 at 1 μM, 50 μM, and 100 μM, or with the anti-malarial drugs ATS and CQ at approximately 50 × EC_50_ for 4 h under cell culture conditions. Subsequently, free thiol groups were blocked with 2 mM *N*-ethylmaleimide (NEM) for 15 min at 37 °C. For 24 h experiments, a 5 mL culture (5% haematocrit, 6–8% parasitaemia) of ring stage parasites (6–10 h post invasion) was treated with ML304 at 1 μM, 5 μM, and 10 μΜ, or the anti-malarial drugs ATS and CQ at approximately 4 × EC_50_. Prior to enrichment, cysteines were blocked with 2 mM NEM for 15 min at 37 °C. For 4 h and 24 h experiments, cells were washed after incubation and resuspended in Ringer’s solution (122.5 mM NaCl, 5.4 mM KCl, 1.2 mM CaCl_2_, 0.8 mM MgCl_2_, 11 mM d-glucose, 25 mM HEPES, 1 mM NaH_2_PO_4_, pH 7.4). 50 μL of cells (1.0 × 10^6^ cells 50^−1^ μL^−1^) were seeded onto poly-l-lysine-coated μ-Slides 18 well (flat) (Ibidi, Martinsried, Germany) and measured using CLSM (confocal laser scanning microscopy) with excitation wavelengths at 405 nm and 488 nm. All experiments included non-treated parasites as controls and both fully reduced and fully oxidized parasites with 10 mM DTT and 1 mM DIA, respectively (2 min incubation), prior to blocking with NEM. Each experiment was carried out three times. Between 10 and 20 microscopy images were taken each time, resulting in at least 30 experimental values per concentration and incubation time. The obtained ratio values were normalized to the control value, which was set to 100. All experiments were carried out within 6 weeks after the appearance of transfectants. Mean and standard error of the mean (SEM) are shown. A one-way ANOVA test with 95% confidence intervals with the Dunnett’s multiple comparison test (GraphPad Prism 5.0) was applied for statistical analysis of significance (*p < 0.05; **p < 0.01; ***p < 0.001).

## Results

### Production, oligomerization, and kinetic characterization of *Pv*G6PD

**Fig. 1:**
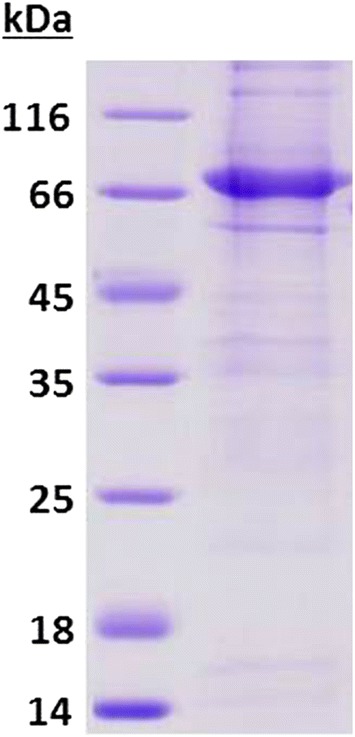
SDS-PAGE and Coomassie staining of purified *Pv*G6PD. The enzyme was heterologously overexpressed in *E. coli* M15 cells containing pRAREII. The protein containing an N-terminal 6×His-tag (71.9 kDa) was purified via Ni–NTA affinity chromatography, followed by size exclusion chromatography. The final purity of *Pv*G6PD was checked via SDS-PAGE (12% acrylamide). Left: unstained protein molecular weight marker (Thermo Scientific, Dreieich, Germany)

Functional *P. vivax* glucose 6-phosphate dehydrogenase, representing the C–terminal part (aa 310–927) of *Pv*GluPho, was recombinantly produced using a pQE30 vector in *E. coli* M15 cells containing the pRAREII plasmid. The yield was 0.1–0.4 mg of active *Pv*G6PD per litre of *E. coli* culture. The final purity of the enzyme was checked via SDS-PAGE (Fig. 1). The recombinant production of the full-length enzyme was attempted in parallel but remained extremely challenging and is therefore not reported in this manuscript. The V_max_ of *Pv*G6PD was determined to be 5.6 ± 0.7 U mg^−1^; the *K*_M_ values were 14.8 ± 2.9 µM for NADP^+^ and 80.2 ± 19.8 µM for G6P. A comparison of the kinetic parameters of *Pv*G6PD to *Pf*G6PD, full-length *Pf*GluPho, and human G6PD is shown in Table 1. The kinetic data of *Pv*G6PD was determined using the standard enzyme assay for *Pf*GluPho according to Jortzik et al. and Beutler [14, 53] with slight modifications.

**Table 1:** Comparison of kinetic data from *Plasmodium vivax* and *Plasmodium falciparum*

	*Pv*G6PD	*Pf*G6PD*	*Pf*GluPho wt	*Pf*GluPho wt*	hG6PD*
V_max_ (U mg^−1^)					
G6P	5.6 ± 0.7	5.5 ± 0.2	5.9 ± 1.0	5.2 ± 1.6	64.4 ± 11
NADP^+^	5.5 ± 0.4	5.5 ± 0.1	5.9 ± 0.6	4.6 ± 0.8	57.7 ± 15
*K*_M_ (μM)					
G6P	80.2 ± 19.8	33.2 ± 1.2	20.3 ± 5.6	19.2 ± 3.9	116 ± 8.5
NADP^+^	14.8 ± 2.9	6.1 ± 0.7	6.1 ± 1.3	6.5 ± 2.2	17.5 ± 2.8
*k*_cat_ (s^−1^)					
G6P	6.7 ± 0.8	6.3 ± 0.3	10.7 ± 1.8	8.6 ± 1.5	64.6 ± 8.9
NADP^+^	6.5 ± 0.4	6.3 ± 0.1	10.1 ± 0.9	8.2 ± 1.2	56.9 ± 15
Catalytic efficiency (L mol^−1^ s^−1^)					
G6P	8.2·10^4^	1.9·10^5^	5.3·10^5^	4.4·10^5^	5.6·10^5^

Values represent mean ± SD of least three independent determinations with different enzyme batches, each including at least two measurements

* Values are taken from Jortzik et al. [14] for comparison. Due to the higher *K*_M_ value for G6P in *Pv*G6PD, 800 µM G6P was defined as substrate saturation. Catalytic efficiency (*k*_cat_/*K*_M_) was calculated from mean values to allow for comparison with the values published therein [14]

Gel filtration chromatography was used to study the oligomerization behaviour of *Pv*G6PD. Under reducing conditions, *Pv*G6PD eluted as a single peak of approximately 389 kDa, indicating that the protein is present as a hexamer (calculated molecular mass of the recombinant His-tagged protein: 71.9 × 6 = 431.4 kDa) and that this oligomerization is not primarily based on intermolecular disulfide bonds.

### Post-translational redox modifications of G6PDs from *Plasmodium falciparum* and *Plasmodium vivax*

**Fig. 2:**
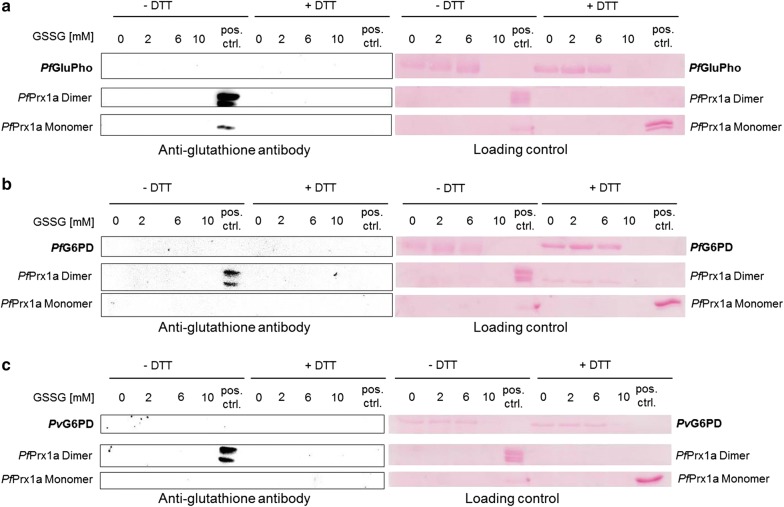
Analysis of *S*-glutathionylation on *Pf*GluPho (**a**), *Pf*G6PD (**b**), and *Pv*G6PD (**c**). Pre-reduced enzymes were incubated with different GSSG concentrations for 10 min at 37 °C; potential *S*-glutathionylation was detected via western blot analysis using an anti-glutathione antibody. To study the reversibility of potential *S*-glutathionylation, reducing SDS-PAGE with DTT was performed. Samples of *Pf*Prx1a were prepared in parallel as a positive control. Without DTT, *Pf*Prx1a exists in a dimeric form; adding DTT shifts the conformation to a monomeric form. Ponceau staining of the membrane used for western blotting served as a loading control. Representative blots from at least two independent experiments are shown. Pos. ctrl.: positive control

*S*-glutathionylation has previously been proposed to regulate the activity of *Pf*GluPho [14]. In this study, pre-reduced *Pv*G6PD was therefore incubated with increasing concentrations of GSSG (up to 10 mM), followed by western blot analysis using anti-glutathione antibodies. *Pf*GluPho and the C-terminal part (*Pf*G6PD) were studied in parallel. Surprisingly, none of the blots showed a signal at the expected height of the proteins (Fig. 2), indicating that neither *Pf*GluPho nor the C-terminal G6PD part of both *P. falciparum* and *P. vivax* were susceptible to redox-regulation via *S*-glutathionylation. It was previously stated that high concentrations of GSSG (8–10 mM) completely inhibit the activity of *Pf*GluPho [14]. After incubation with 10 mM GSSG, however, precipitation of all three enzymes was observed, which also resulted in a lack of the protein bands in the Ponceau-stained membranes (Fig. 2). Therefore, this loss of activity is likely based on an irreversible loss of conformation rather than on redox regulation.

**Fig. 3:**
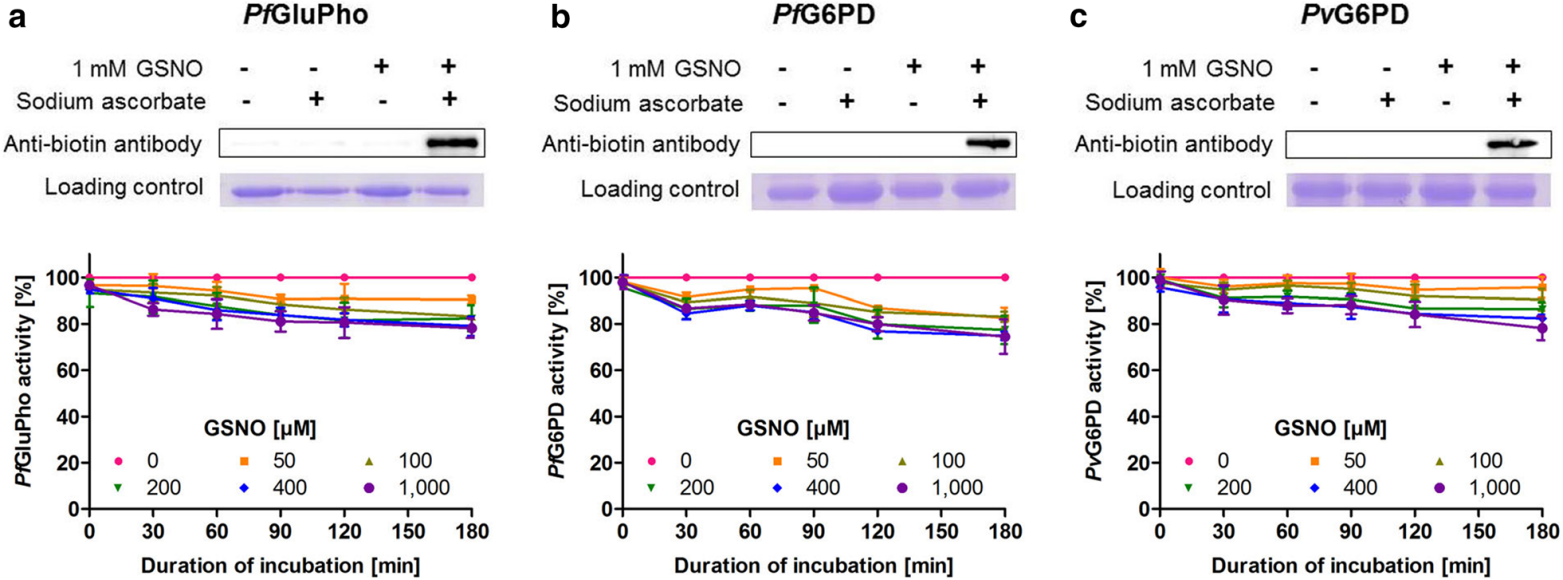
Analysis of *S*-nitrosation on *Pf*GluPho (**a**), *Pf*G6PD (**b**), and *Pv*G6PD (**c**). Pre-reduced enzymes were incubated with 0 or 1 mM GSNO for 1 h at 22 °C in the dark and subjected to the biotin switch assay. Samples without sodium ascorbate served as a control. *S*-nitrosylated proteins were detected by anti-biotin western blot. Coomassie staining of the samples used for western blotting served as a loading control. Representative blots from at least two independent experiments are shown. To study the impact of *S*-nitrosation on enzyme activity, pre-reduced enzymes were incubated with different concentrations of GSNO (0–1000 µM) for 180 min at 22 °C in the dark. Activity of each sample was measured directly after adding GSNO, as well as after 30, 60, 90, 120, and 180 min. Activity of the sample with 0 µM GSNO was defined as 100%. Values shown are mean ± SD from at least three independent determinations using two different batches of enzyme

To identify potential *S*-nitrosation in *Pf*GluPho/*Pf*G6PD as well as *Pv*G6PD, pre-reduced enzymes were incubated with 1 mM GSNO and afterwards subjected to the biotin-switch assay [41]. GSNO is a physiologically relevant *S*-nitrosated derivative of glutathione capable of transnitrosating proteins [54]. Western blot analyses clearly showed a signal at the expected height for the samples treated with 1 mM GSNO and NaAsc. In contrast, control samples without NaAsc or without GSNO showed no signal (Fig. 3).

To investigate the impact of *S*-nitrosation on the activity of the enzymes, the pre-reduced proteins were incubated with different concentrations of GSNO (0–1000 µM), and their remaining activities were measured at several time points. Incubation with 1000 µM GSNO for 3 h resulted in an inhibition of only 20–25%; lower concentrations and shorter incubation times resulted in even lower inhibition, indicating that *Pf*GluPho, its C-terminal part *Pf*G6PD, and *Pv*G6PD as the C-terminal part of *Pv*GluPho are most likely not extensively regulated via *S*-nitrosation (Fig. 3). This is further underscored by the fact that adding DTT as a reducing agent was not able to restore the enzyme activities completely.

### *Kinetic characterization of naturally occurring* Pf*GluPho variants*

There are several naturally occurring variants of *Pf*GluPho. These variants (single nucleotide polymorphisms) were identified via a detailed in silico analysis of the (former) ID: PF14_0511 (now PF3D7_1453800) in the malaria database PlasmoDB. The most prominent SNPs in the G6PD part of the enzyme leading to an amino acid exchange were S315Y, L395F, and F507L. So far, there have been no investigations on whether these mutations have an impact on enzymatic activity. Furthermore, several amino acid residues (including S327, Y329, S330, S332, S477, T492, S701, S899, and S900) of *Pf*GluPho were found to be phosphorylated in the parasites. Interestingly, the presence of the C-terminally located Ser–Ser pair (S899 and S900) was found to be specific for and highly conserved in *Plasmodium* species. Furthermore, the phosphorylation of these two residues was consistently identified by several groups [36–38] (for sequence alignment with G6PDs from different species, please see Additional file 1). To study whether the naturally occurring variants have an impact on *Pf*GluPho activity, the respective mutations were inserted into the recombinant protein. To mimic *S*-phosphorylation as described in Kim et al. [39], Ser → Glu mutations were inserted at aa positions 899 or 900. After purification and size exclusion chromatography under reducing conditions, all mutants eluted as tetramers, indicating that the oligomerization is not based on disulfide bonds between the subunits and was not disturbed by the mutations. This is in accordance with the literature for the wt enzyme [14]. The yield of pure and active *Pf*GluPho mutants varied from 0.3 to 2.2 mg per liter *E. coli* culture.

An overview of the kinetic data is given in Table 2. The values for the wt enzyme are in accordance with previously reported data [14]. The V_max_ values of the enzyme mutants ranged between 4.0 and 6.0 U mg^−1^ and did not show any major differences from the wt enzyme (5.9 ± 1.0 U mg^−1^ for G6P, 5.9 ± 0.6 U mg^−1^ for NADP^+^, see also [14]). The *K*_M_ values were in the range of 18.4 and 29.2 µM for G6P (wt: 20.3 ± 5.6 µM, see also [14]) and of 3.7 and 6.2 µM for NADP^+^ (wt: 6.1 ± 1.3 µM, see also [14]). This indicates that neither the naturally occurring mutants of the G6PD part of *Pf*GluPho nor the mimicked phosphorylation of the two C-terminally located serine residues have a major impact on the catalytic activity of the enzyme. Further experiments using phosphorylated *Pf*GluPho need to be performed to confirm these results.

**Table 2:** Kinetic characteristics of *P. falciparum* GluPho variants

	*Pf*GluPho wt*	*Pf*GluPho wt	*Pf*GluPho^S315Y^	*Pf*GluPho^L395F^	*Pf*GluPho^F507L^	*Pf*GluPho^S899E^	*Pf*GluPho^S900E^
V_max_ (U mg^−1^)							
G6P	5.2 ± 1.6	5.9 ± 1.0	4.4 ± 1.1	4.5 ± 0.9	6.0 ± 1.4	5.6 ± 0.1	6.0 ± 1.1
NADP^+^	4.6 ± 0.8	5.9 ± 0.6	4.6 ± 1.5	4.0 ± 1.1	5.0 ± 0.3	5.2 ± 0.2	6.0 ± 0.6
*K*_M_ (μM)							
G6P	19.2 ± 3.9	20.3 ± 5.6	23.4 ± 7.1	29.2 ± 4.1	24.1 ± 4.4	24.1 ± 2.8	18.4 ± 5.1
NADP^+^	6.5 ± 2.2	6.1 ± 1.3	5.1 ± 1.0	4.0 ± 0.2	3.7 ± 0.1	6.2 ± 0.5	5.8 ± 1.5
*k*_cat_ (s^−1^)							
G6P	8.6 ± 1.5	10.7 ± 1.8	7.9 ± 2.0	8.1 ± 1.6	10.9 ± 2.5	10.1 ± 0.2	10.8 ± 1.9
NADP^+^	8.2 ± 1.2	10.1 ± 0.9	8.3 ± 2.7	7.2 ± 2.0	9.1 ± 0.5	9.3 ± 0.4	10.8 ± 1.2
Catalytic efficiency (L mol^−1^ s^−1^)							
G6P	4.4·10^5^	5.8·10^5^ ± 1.5∙10^5^	3.7·10^5^ ± 2.0∙10^5^	2.9·10^5^ ± 9.4∙10^4^	4.5·10^5^ ± 2.2∙10^4^	5.2·10^5^ ± 2.3∙10^5^	6.2∙10^5^ ± 2.2∙10^5^

Values represent mean ± SD of at least two independent determinations with different enzyme batches, each including at least two independent measurements

* Values are from Jortzik et al. [14] for comparison. Catalytic efficiency (*k*_cat_/*K*_M_) from there [14] was calculated from mean values to allow for comparison

### Inhibition of *Pv*G6PD with ellagic acid and derivatives

**Fig. 4:**
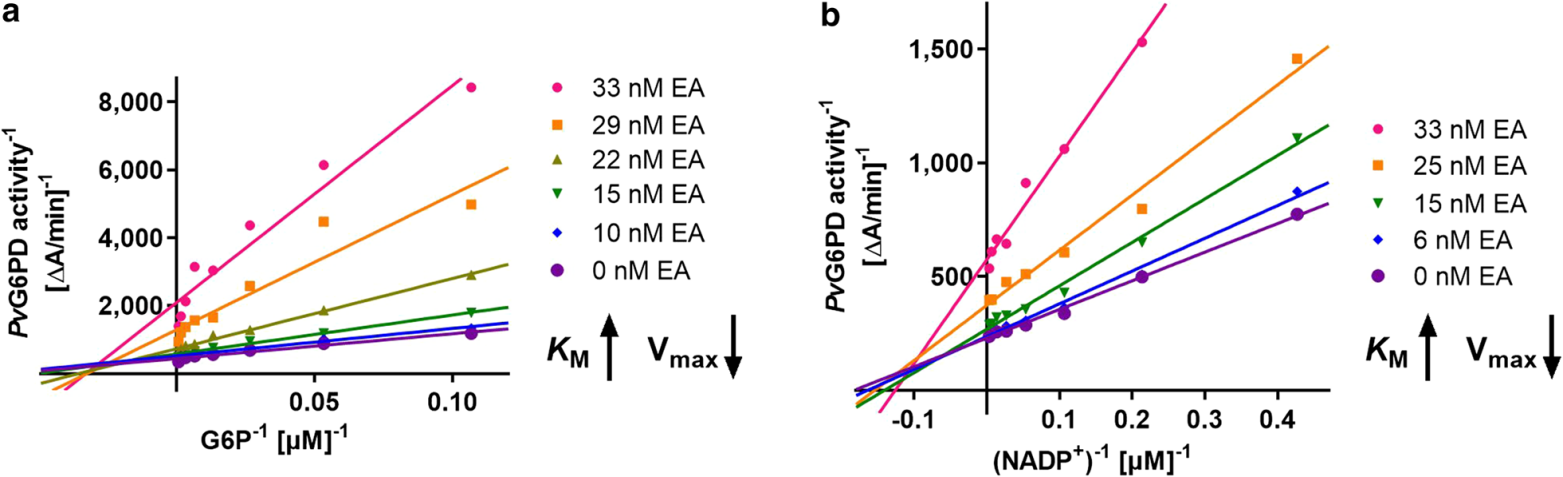
Inhibition of *Pv*G6PD with ellagic acid. The Lineweaver–Burk plots for titrating G6P (**a**) or NADP^+^ (**b**) show that the higher the concentration of ellagic acid, the lower the V_max_, while the *K*_M_ values increased. This indicates that ellagic acid acts as a mixed-type inhibitor of *Pv*G6PD with respect to both G6P and NADP^+^. Representative graphs from at least four independent determinations are shown

As previously reported, ellagic acid, a polyphenolic lactone extracted from plants used in traditional medicine in Africa, reversibly inhibits *Pf*GluPho with an IC_50_ of 77 ± 22 nM [15]. In this study, ellagic acid is shown to also inhibit the *P. vivax* enzyme *Pv*G6PD with an IC_50_ of 32.5 ± 13.4 nM. Mechanistic characterization indicated that ellagic acid acts as a mixed type inhibitor against both G6P and NADP^+^ in *Pv*G6PD, which is identical to the mechanism determined for the *P. falciparum* enzyme [15]. With increasing EA concentrations, the *K*_M_ values for both substrates increased, while V_max_ decreased (Fig. 4).

In addition to EA, the effects of two EA derivatives, FEA and CEA, on *Pv*G6PD were determined. FEA inhibited *Pv*G6PD with an IC_50_ of 37.2 ± 5.8 nM, and CEA showed an IC_50_ of 44.4 ± 1.9 nM.

### ML304, an inhibitor selective for *Plasmodium* G6PD

**Fig. 5:**
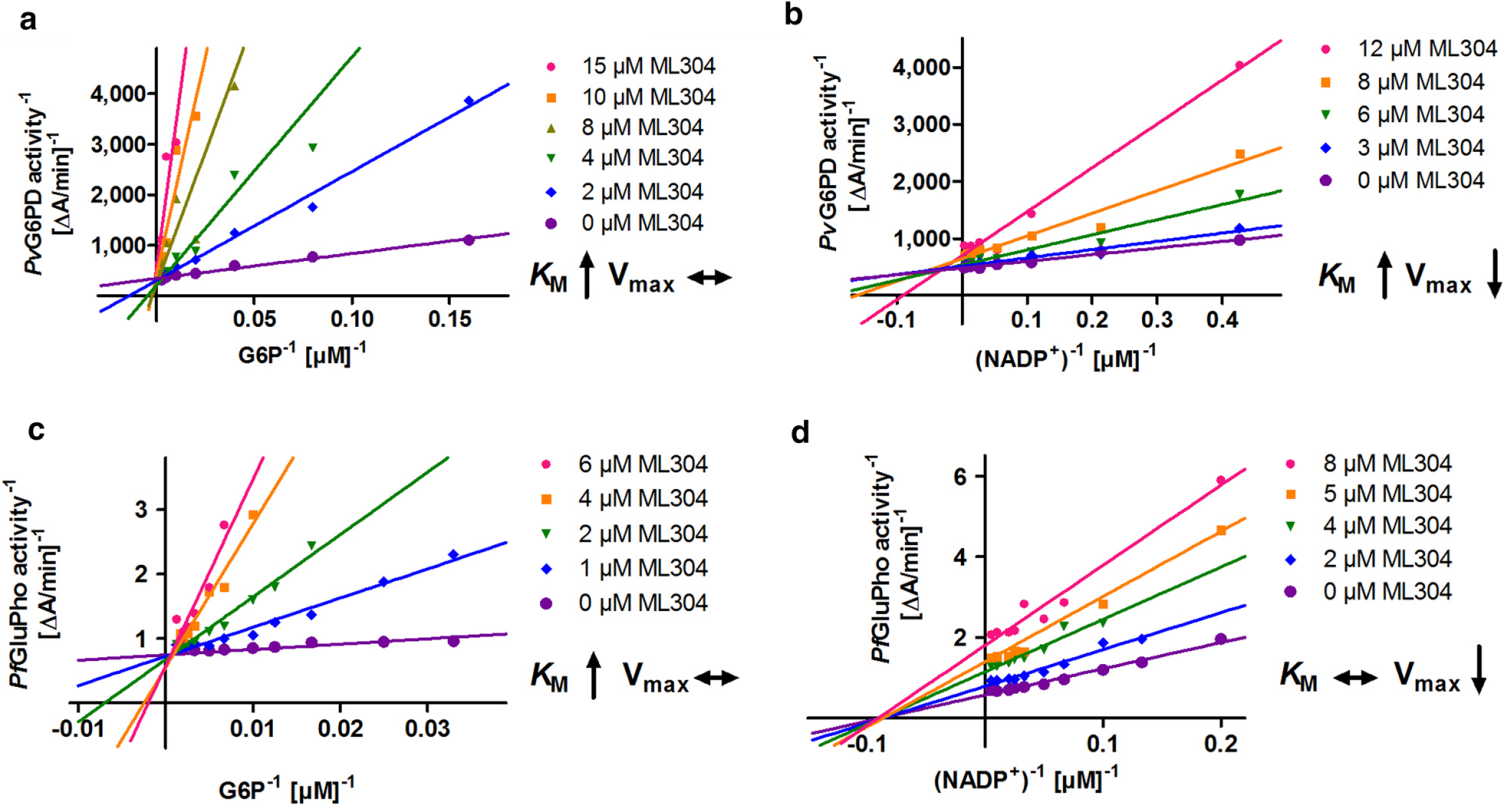
Inhibition of *Pv*G6PD (**a**, **b**) and *Pf*GluPho (**c**, **d**) by ML304. The data indicates a competitive inhibition with respect to G6P for both enzymes. The higher the concentration of ML304, the higher the *K*_M_ values of *Pv*G6PD and *Pf*GluPho for G6P were; V_max_ stayed constant (**a**, **c**). With respect to NADP^+^, ML304 acts as a mixed type inhibitor on *Pv*G6PD (**b**) and as a non-competitive inhibitor on *Pf*GluPho (**d**)

Via a high-throughput screening of the MLSMR chemical library, the compound ML304 was identified as a selective inhibitor of the G6PD activity of *Pf*GluPho. The IC_50_ was found to be 190 nM on the *P. falciparum* enzyme and 80 µM on human G6PD [18]. Under conditions of substrate saturation (800 µM G6P, 200 µM NADP^+^), ML304 was found to inhibit *Pv*G6PD with an IC_50_ of 15.3 ± 0.9 µM. Application of substrate and coenzyme close to the *K*_M_ values of *Pv*G6PD (85 µM G6P, 15 µM NADP^+^) resulted in a lower IC_50_ of 2.6 ± 0.8 µM. This was confirmed by mode of inhibition studies, which showed that ML304 acts as a competitive inhibitor towards G6P (*K*_i_ = 0.7 ± 0.3 µM) and as a mixed type inhibitor towards NADP^+^ (*K*_i_ = 16.3 ± 8.8 µM) (Fig. 5a, b) [55]. On *Pf*GluPho, ML304 also competes with G6P (*K*_i_ = 0.23 ± 0.16 µM). Towards NADP^+^, it acts as a non-competitive inhibitor (Fig. 5c, d).

### ML304 affects the cytosolic redox potential of *P. falciparum* blood stage parasites

**Fig. 6:**
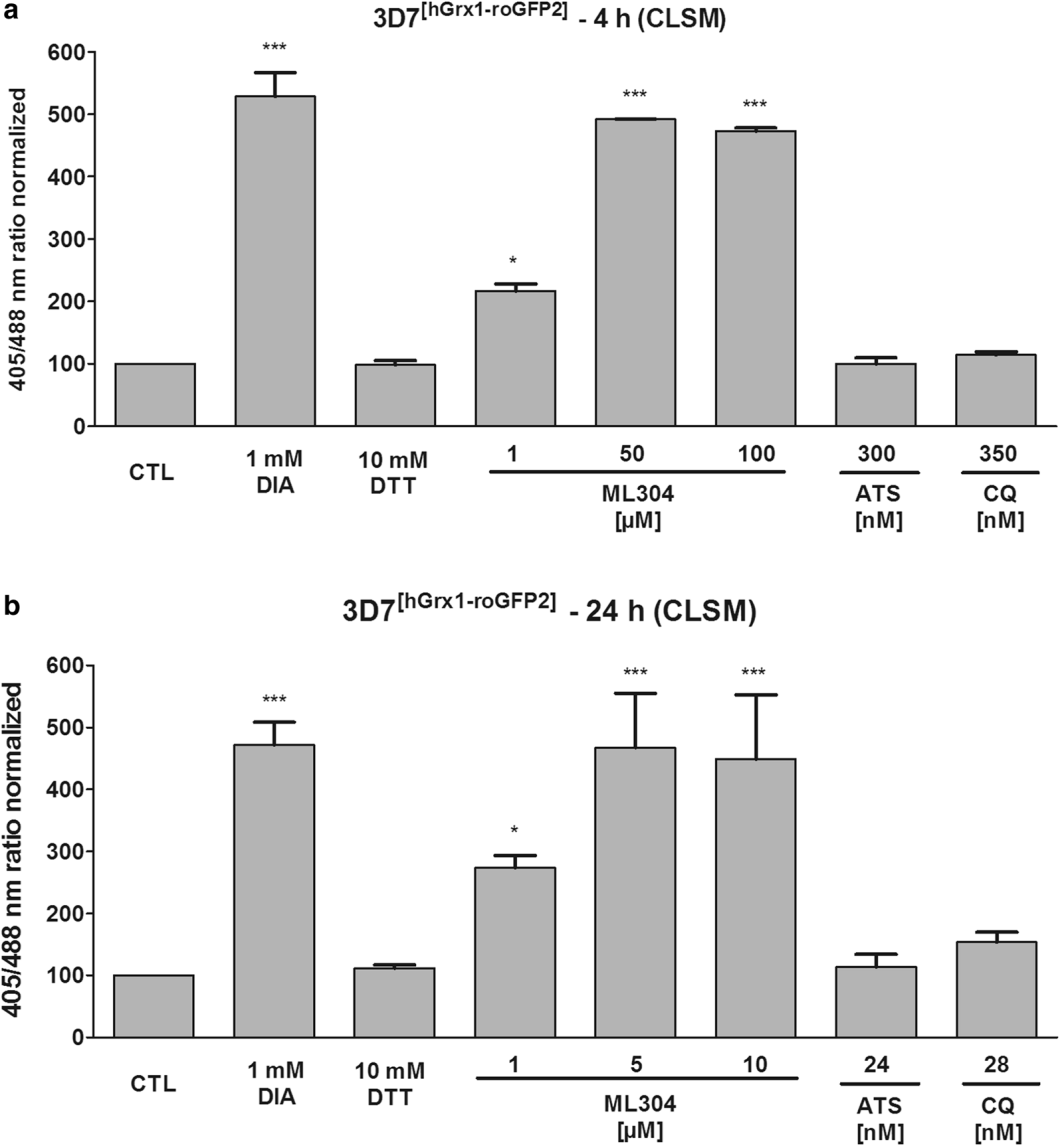
Mid- and long-term effects of ML304 on the redox ratio of *P. falciparum* 3D7^[hGrx1-roGFP2]^ parasites. *Plasmodium falciparum* cells were treated with ML304 at 1 µM, 50 µM, and 100 µM for 4 h (**a**). For 24 h incubations (**b**), cells were treated with ML304 at 1 µM, 5 µM, and 10 µM. The anti-malarial drugs ATS and CQ were also tested in both 4 h and 24 h experiments for comparison. Free thiols were subsequently blocked with 2 mM NEM. Both 4 h and 24 h incubations of 3D7^[hGrx1–roGFP2]^ parasites with ML304 showed significant increases in the 405/488 nm fluorescence ratio using CLSM. Non-treated parasites served as controls. All experiments included fully oxidized (1 mM DIA) and fully reduced (10 mM DTT) parasites (each using 2 min incubations) prior to blocking with NEM. CLSM data was obtained from 10 to 20 trophozoites for each experiment and each incubation time. Mean values and standard errors of the means (± SEM) are shown for three independent experiments. A one-way ANOVA test with 95% confidence intervals with the Dunnett’s multiple comparison test was applied for statistical analysis of significance (*p < 0.05; **p < 0.01; ***p < 0.001) and indicated significant changes compared to the respective control

ML304 [18] has recently been described to strongly impair the viability of *Plasmodium* gametocytes, the sexual forms of the parasite that can be transmitted to the *Anopheles* vector [56]. To further characterize the biological effects of the G6PD inhibitor ML304, its influence on the cytosolic glutathione-dependent redox potential was determined. For this, the genetically encoded redox sensor hGrx1–roGFP2 (consisting of human glutaredoxin 1 coupled to redox-sensitive green fluorescent protein) was transiently expressed in the cytosol of transfected *P. falciparum* 3D7 parasites. Mid- and long-term exposures were carried out via confocal laser scanning microscopy (CLSM). Control studies excluded a direct interaction of ML304 and the drugs ATS and CQ with the recombinantly purified hGrx1–roGFP2 probe at the concentrations used in this study. 3D7^[hGrx1–roGFP2]^-enriched trophozoites were treated with different concentrations of ML304 and the anti-malarial drugs ATS and CQ based on their EC_50_ values [47] for 4 h and 24 h under cell culture conditions, and reactions were subsequently blocked with 2 mM NEM. The EC_50_ value of ML304 on *P. falciparum* 3D7 determined in the 72 h hypoxanthine incorporation assay was confirmed to be in the mid-nanomolar range (471.3 ± 39.5 nM). Figure 6a shows that ML304 had significant effects on 3D7^[hGrx1–roGFP2]^ parasites at all concentrations used in the 4 h incubation experiments (p < 0.05 at 1 µM, p < 0.001 at 50 µM and 100 µM). In 24 h experiments (Fig. 6b), ML304 also significantly increased the 405/488 nm ratio of the hGrx1-roGFP2 sensor (p < 0.05 at 1 µM, p < 0.001 at 50 µM and 100 µM). 50 µM of ML304 in 4 h incubations seemed to completely oxidize the sensor as 5 µM of ML304 in 24 h incubations did, which was comparable to the treatment with 1 mM DIA. Therefore, higher concentrations above 50 µM (4 h) and 5 µM of ML304 (24 h) did not increase oxidation of the probe. 24 h incubation with ATS and CQ hardly affected the 405/488 nm ratio at the concentrations used in the experiment. Only CQ increased the redox ratio 1.5-fold in the 24 h incubations (Fig. 6b).

**Fig. 7:**
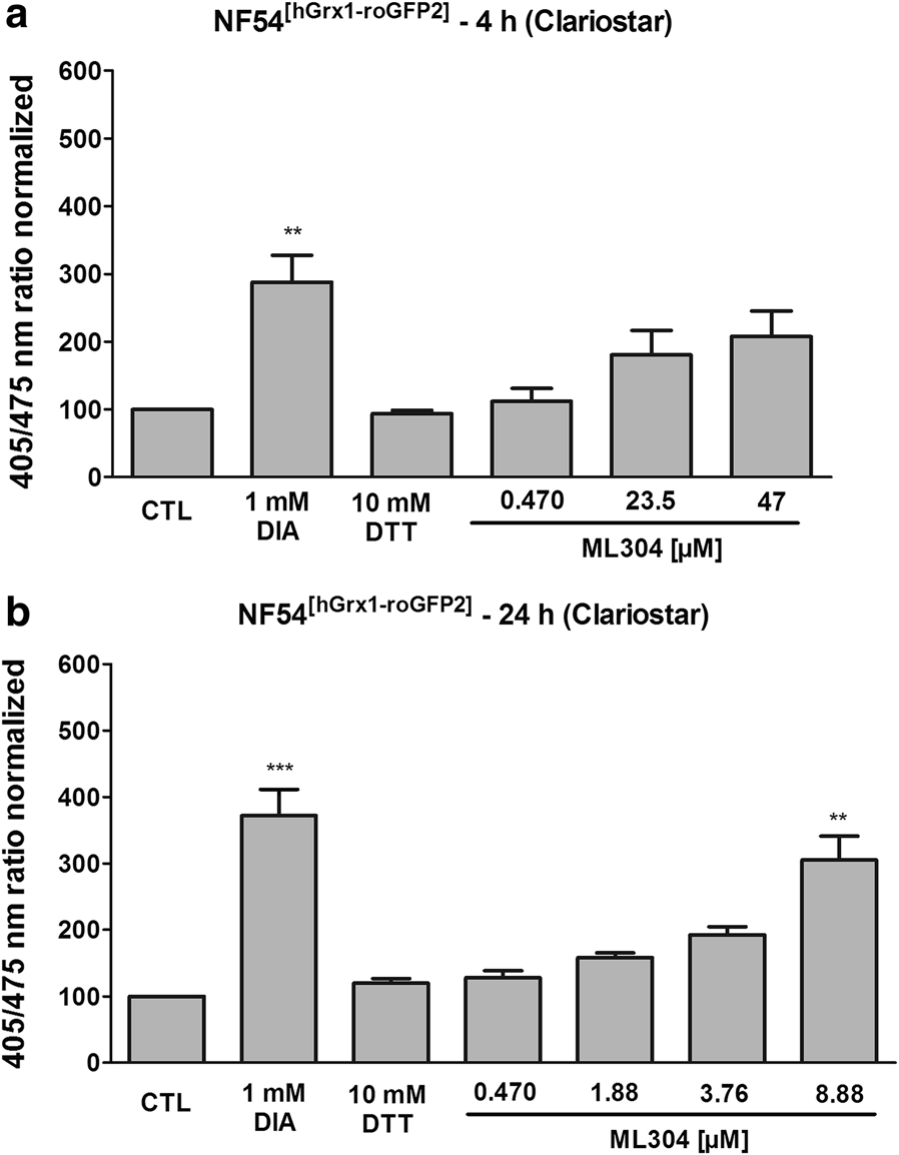
Mid- and long-term effects of ML304 on the glutathione redox ratio of *Plasmodium falciparum* NF54^[hGrx1–roGFP2]^. 4 h incubation of NF54^[hGrx1–roGFP2]^ -transfectants with ML304 led to a concentration-dependent increase in fluorescence ratio as detected using the Clariostar plate reader (**a**). 24 h incubation with varying ML304 concentrations led to increases in 405/475 nm fluorescence ratios with a significant effect of the highest compound concentration (8.88 µM) (**b**). Non-treated parasites served as controls. All experiments included fully oxidized (1 mM DIA) and fully reduced (10 mM DTT) parasites (each using 2 min incubations) prior to blocking with NEM. Mean values and standard errors of the means (± SEM) are shown for three independent experiments. A one-way ANOVA test with 95% confidence intervals with Dunnett’s multiple comparison test was applied for statistical analysis of significance (**p < 0.01; ***p < 0.001)

Recently, the cytosolic glutathione redox sensor was stably integrated into NF54-*attB* parasites [52]. The direct response of NF54^[hGrx1–roGFP2]^ to mid- and long-term exposures were determined using the time-efficient plate reader (Clariostar). Cells were incubated with 1 to 100 times EC_50_ ML304 for 4 h at the trophozoite stage or with approx. 1 to 20 times EC_50_ for 24 h at the ring stage, and reactions were subsequently blocked with 2 mM NEM. As depicted in Fig. 7, ML304 oxidized the cytosol, leading to an increase of the 405/488 nm ratio of the probe in a concentration-dependent manner with significant differences after a 24 h incubation. Treatment with 8.88 µM ML304 for 24 h significantly increased the 405/475 nm fluorescence ratio (threefold, p < 0.01).

### Discovery of novel G6PD inhibitors

**Fig. 8:**
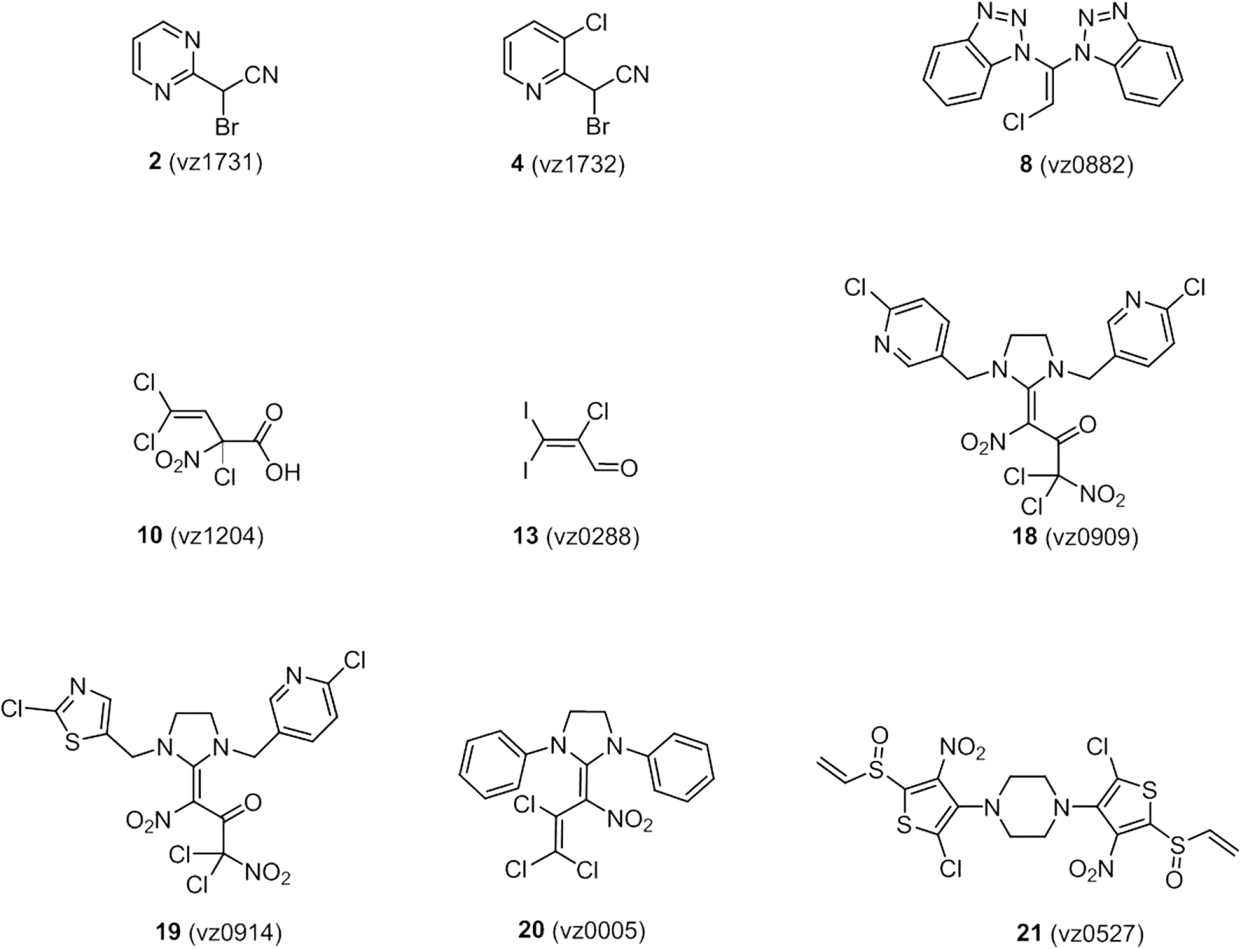
Structures of the most active newly synthesized G6PD inhibitors

In order to identify potential novel inhibitors of *Plasmodium* G6PD, several libraries of the Helmholtz Center for Infection Research, Brunswick, were screened against *Pf*GluPho using the high-throughput-compatible assay systems described in Preuss et al. [16, 17]. The compound library developed by DK and VZ (Clausthal University of Technology) and distributed by Florenz Sasse (Helmholtz Center for Infection Research) centres on the downstream chemistry of the synthetic key units trichloronitroethylene and 2-nitroperchloro-1,3-butadiene. Based on structure–activity relationships of the PASS system (Prediction of Activity Spectra for Substances), the general biological potential of an organic drug-like molecule could be predicted. A set of new potential G6PD inhibitors was then synthesized and assessed (Fig. 8).

**Fig. 9:**
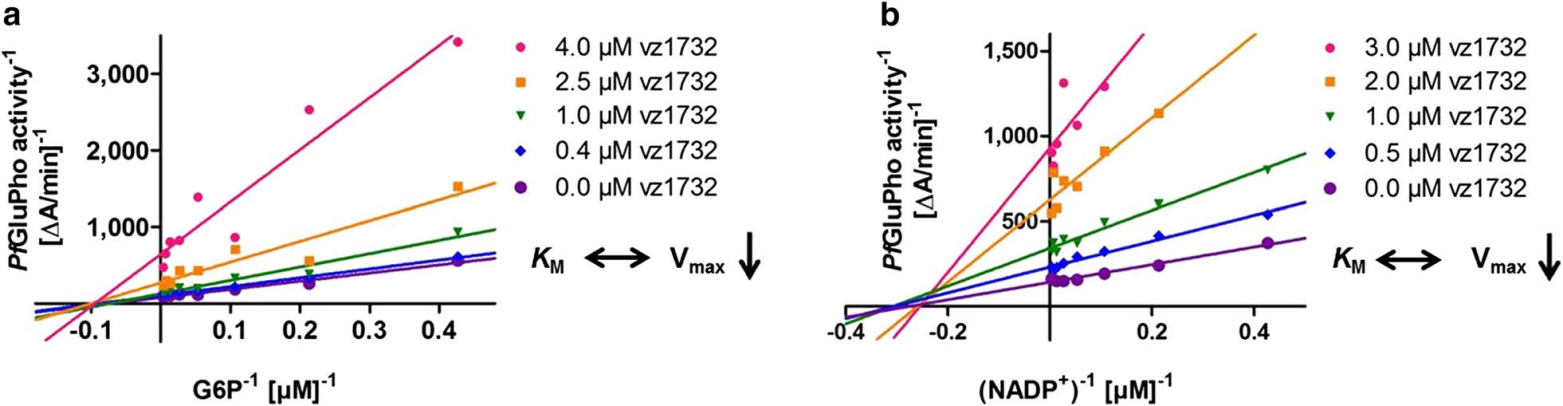
Inhibition type of 4 (vz1732) towards *Pf*GluPho. The compound shows non-competitive inhibition against both G6P (**a**) and NADP^+^ (**b**). The higher the concentration of 4 (vz1732), the lower the V_max_, while the *K*_M_ values for G6P and NADP^+^ stayed constant. Representative graphs from two independent determinations are shown

All compounds were tested on *Pf*GluPho, *Pv*G6PD, and human G6PD (Table 3). **21** (vz0527) and **4** (vz1732) were the most potent compounds, with IC_50_s reaching the nanomolar range. The IC_50_ values of the two compounds on hG6PD were one to two orders of magnitude higher, indicating that a selective targeting of the plasmodial enzymes is possible. The partially high standard deviations were due to limited solubility or stability of the compounds. In order to further characterize the most potent, but structurally rather simple, compound **4** (vz1732), its mode of action against *Pf*GluPho was determined. The compound was found to act non-competitively against both the substrate G6P and the coenzyme NADP^+^ (Fig. 9), indicating that the compound does not interact with either of the two binding sites.

**Table 3:** IC_50_ values of newly synthesized inhibitors on *Pf*GluPho, *Pv*G6PD, and hG6PD

	*Pf*GluPho [µM]	*Pv*G6PD [µM]	hG6PD [µM]
**2** (vz1731)	11.7 ± 9.9	2.1 ± 0.1	91.7 ± 35.0
**4** (vz1732)	0.9 ± 0.2	0.2 ± 0.0	34.7 ± 21.0
**8** (vz0882)	18.1 ± 10.4	12.8 ± 7.8	N. d.
**10** (vz1204)	N. d.	23.5 ± 3.7	> 100
**13** (vz0288)	29.6 ± 23.2	23.3 ± 7.8	> 100
**18** (vz0909)	23.6 ± 14.3	9.1 ± 2.8	26.0 ± 1.8
**19** (vz0914)	8.4 ± 8.4	5.6 ± 0.6	29.6 ± 5.0
**20** (vz0005)	> 100	> 100	> 100
**21** (vz0527)	1.7 ± 0.3	0.2 ± 0.1	8.3 ± 2.7

*N. d.* not determined due to low solubility/reproducibility

Values are represented as mean ± SD of at least two independent determinations using different batches of enzyme, each including at least two independent measurements. For hG6PD, only one enzyme batch was used

## Discussion

The key enzyme of the oxidative PPP of *P. falciparum*—*Pf*GluPho—has been shown to be essential for blood stage parasites and is a potential target for the development of new anti-malarial drugs [14, 15]. The corresponding enzyme of *P. vivax*—*Pv*GluPho—also exists as a bifunctional enzyme and shares high structural and functional similarity with *Pf*GluPho (71.2% identity and 79.9% similarity on aa level, Additional file 5). In this study, the cloning, heterologous overexpression, and purification of *Pv*G6PD, representing the C-terminal part of *Pv*GluPho, is described. When comparing the kinetic parameters of *Pf*GluPho and *Pv*G6PD, higher *K*_M_ values for substrate and cofactor became evident for the *P. vivax* enzyme, indicating that the *P. falciparum* enzyme is likely to have advantages when competing with the human host G6PD for substrates. Whether this fact contributes to the higher pathogenicity of *P. falciparum* deserves to be studied in more detail. Interestingly, the *K*_M_ for G6P of *Pf*G6PD (33.2 ± 1.2 µM [14]) is higher than the *K*_M_ of the full-length enzyme *Pf*GluPho (19.2 ± 3.9 µM), and the catalytic efficiency is lower (1.9 × 10^5^ L mol^−1^ s^−1^ for *Pf*G6PD, 4.4 × 10^5^ L mol^−1^ s^−1^ for *Pf*GluPho, see [14]). This might be explained by structural advantages of the fusion protein, which plays a central role in the parasites’ metabolism [57]. This notion might also be true for the *P. vivax* enzyme; the fusion of G6PD with 6PGL in full-length *Pv*GluPho and the potentially altered oligomerization state of the protein might result in lower *K*_M_ values and a higher catalytic activity. Confirming measurements using the full-length enzyme need to be conducted as soon as it is available. Interestingly, however, V_max_ and corresponding k_cat_ values are one order of magnitude higher in hG6PD than in the plasmodial enzymes, resulting in a higher catalytic efficiency of the human enzyme (Table 1). This underscores the importance of G6PD for red blood cells and might be (partially) compensated in the parasites by the fusion of G6PD with the second enzyme of the PPP, allowing for direct substrate transfer within the pathway.

G6PDs from most organisms exist as monomers (*Bos taurus* [58]), dimers (e.g. *Trypanosoma brucei* [59]), or tetramers (*Sus scrofa* [60]); hG6PD exists in all three forms depending on the ionic strength, pH, and presence of substrates [61] and under native and reducing conditions, *Pf*GluPho is active as a tetramer [14]. In this study, recombinant *Pv*G6PD was present as a hexamer under reducing conditions. Such a hexameric oligomerization was not previously described for G6PDs (Brenda, EC 1.1.1.49) and might actually represent an artificial state since *Pv*G6PD occurs as a fusion protein with 6PGL under physiological conditions. The oligomerization state of full-length *Pv*GluPho is presumably different—based on the high sequence identity with *Pf*GluPho likely tetrameric.

### Post-translational redox modifications of G6PDs from *Plasmodium falciparum* and *Plasmodium vivax*

In recent years, the importance of enzyme regulation via post-translational modification (PTM) of reactive cysteine residues has been increasingly recognized [62, 63]. Therefore, two of the most important PTMs, protein *S*-glutathionylation and *S*-nitrosation, were studied on G6PD of *P. falciparum* and *P. vivax*. *S*-glutathionylation has been shown to play a role in protein folding and stability as well as the redox regulation of multiple metabolic pathways. During episodes of oxidative stress, *S*-glutathionylated proteins can serve as a storage form of GSH, while the modification prevents the protein from irreversible oxidation [64]. As indicated in Fig. 2, neither *Pf*GluPho nor *Pf*G6PD and *Pv*G6PD are prone to *S*-glutathionylation at concentrations up to 6 mM GSSG. At 10 mM GSSG the proteins precipitated irreversibly. Therefore, the previous hypothesis that the activity of *Pf*GluPho decreases after incubation with higher GSSG concentrations [14] is likely to represent an artefact rather than a specific regulation. This is also in accordance with previous proteomic analyses, where *Pf*G6PD was not identified as a target of *S*-glutathionylation [40]. As recently reported, 6-phosphogluconate dehydrogenase is not susceptible to *S*-glutathionylation either [42], suggesting that this redox PTM does not play a major role in the regulation of the oxidative PPP in *Plasmodium*.

Protein *S*-nitrosation mediates many (patho)physiological effects of nitric oxide (NO). It can influence protein conformation, stability, and activity; alter protein–protein interactions; and influence cellular signal transduction processes [62, 65]. In a previous proteomic approach, *Pf*GluPho was identified as a target of *S*-nitrosation [41], which could be confirmed—for *Pf*GluPho, *Pf*G6PD, and *Pv*G6PD—in this study. Since the C-terminal part of *Pf*GluPho, *Pf*G6PD, showed the same results as full-length *Pf*GluPho in both western blot and kinetic analyses, *S*-nitrosation sites are likely located in the G6PD part of the enzyme. *S*-nitrosation of all three enzymes leads only to minor changes in enzyme activity (Fig. 3).

### Kinetic characterization of *Pf*GluPho variants

During recent years, extensive research has been performed to elucidate the genome-wide diversity of *Plasmodium* species [31]. These variants might supply the parasites with evolutionary benefits to deal with the selection pressure [30]. Interestingly, there are also naturally occurring mutations within the *Pf*GluPho gene. Since this enzyme has a central role in the maintenance of cellular redox balance, it is hypothesized that these mutations might lead to altered enzymatic activity and modulation of antioxidant defense. Therefore, the mutants *Pf*GluPho^L395F^ and *Pf*GluPho^F507L^, which are located in the G6PD domain of the *Pf*GluPho gene, as well as *Pf*GluPho^S315Y^ located in the linker domain between 6PGL and G6PD, were created, produced, and tested. F507 is located in an insertion sequence of the gene that is specific for G6PDs of *Plasmodium* and differs in size and sequence between the species. In the *Plasmodium berghei* enzyme, this insertion has even been shown to be essential for G6PD activity [66]. However, neither enzymatic activity nor substrate affinities of the tested mutants differed significantly from the wt enzyme (Table 2). Moreover, the potential impact of the naturally occurring phosphorylation was assessed (for a review see [67]) of the *Plasmodium*-specific serine residues 899 and 900 on the kinetic parameters of *Pf*GluPho (Additional file 1). However, the respective phosphorylation-mimicking mutants did not show significant differences to the wt. Therefore, it can be concluded that the activity of *Pf*GluPho is not strongly regulated in vivo by the three naturally occurring mutations or the phosphorylation of serines 899 and 900, unless these variations influence other properties of the enzyme such as protein–protein interaction or stability.

### G6PD as a drug target

Within this study, various potential inhibitors were tested on *Pv*G6PD, namely ellagic acid, its derivatives flavellagic acid and coruleoellagic acid, the *Pf*GluPho inhibitor ML304, and a new series of compounds synthesized as potential *Plasmodium* G6PD inhibitors.

#### Ellagic acid

Ellagic acid was identified to be an active component in plants that were used in traditional West African medicine for the treatment of malaria [32]. Interestingly, it inhibits the growth of *P.* *falciparum* strains in vitro (IC_50_s from 105 nM to 1.3 nM depending on the strain) and those that are resistant to chloroquine and mefloquine [32, 33]. Additionally, it is active against *Plasmodium vinckei petteri* in an in vivo mouse model (IC_50_ 1 mg kg^−1^ per day i.p.) in suppressive, curative, and prophylactic tests while being well tolerated [32]. Ellagic acid is supposed to act against mature trophozoites and young schizonts and shows synergistic effects with chloroquine, atovaquone, mefloquine, and artesunate [32]. The mechanism of action of EA has been discussed extensively in several studies. Different enzymes such as glutathione reductase, glutathione *S*-transferase, and thioredoxin reductase are known to be targeted by the compound and its derivatives FEA and CEA, (IC_50_ between 22 and 78 µM [33, 34]). Furthermore, EA is supposed to decrease the glutathione pool; this observation is strengthened by the finding that a *Plasmodium yoelii* strain overproducing GSH is five times less sensitive towards EA than the wt [68]. In summary, the observations so far have indicated a rather broad mechanism of action by affecting the antioxidant balance of the parasite. The authors recently showed that EA inhibits *Pf*GluPho with an IC_50_ of 77 ± 22 nM, which is three orders of magnitude lower than the IC_50_ values determined for other redox-related enzymes [15]. As shown in this study, EA also strongly inhibits *Pv*G6PD (IC_50_ = 32.5 ± 13.4 nM), and the mixed type inhibition with respect to both G6P and NADP^+^ as observed for *Pf*GluPho could be confirmed for *Pv*G6PD (Fig. 4). The IC_50_ values of the EA derivatives FEA and CEA on *Pv*G6PD were also in the lower nanomolar range. Although the effect of EA is not selective for plasmodial enzymes (IC_50_ for hG6PD: 102 ± 39 nM) [15], the overall toxicity in mice is low, and there are no observable haemolytic effects [32], pointing to a higher susceptibility of the rapidly growing and multiplying parasite.

#### ML304

The compound ML304 was identified during a high-throughput screening of various compound libraries including LOPAC, Spectrum, DIVERSet, and the MLSMR collection of the NIH [16]. Two compounds of the MLSMR collection were identified to be potent inhibitors of the G6PD activity of *Pf*GluPho, namely ML276 and ML304. Synthesis and detailed enzymatic and pharmacokinetic characterization of ML276 is described in [17]. With an IC_50_ of 0.89 µM against *Pf*GluPho and of over 80 µM against hG6PD, ML276 is selective for the plasmodial enzyme [17]. ML304, so far only described in an NIH probe report, has an IC_50_ of 190 nM against *Pf*GluPho (> 80 µM against hG6PD) and is even more effective than ML276 [18]. In cell culture, ML304 is active on the chloroquine-sensitive strain *P. falciparum* 3D7 at concentrations lower than 1 µM and on the CQ resistant strain Dd2 at a concentration of about 5 µM. With an LD_50_ > 50 µM, major cytotoxicity against human hepatocyte cell lines could be ruled out [18].

Within this study, ML304 was also confirmed to be an inhibitor of *Pv*G6PD with an IC_50_ of 2.6 ± 0.8 µM (at substrate concentrations close to *K*_M_ values) and a *K*_i_ value of 0.7 ± 0.3 µM. Mechanistic studies indicated a competition with the substrate G6P (Fig. 5a). In cell biological experiments, it could furthermore be shown that ML304 significantly disturbs the cytosolic glutathione-dependent redox potential of *P. falciparum* blood stage parasites. This was shown for 3D7 parasites episomally transfected with the hGrx1–roGFP2 redox sensor (Fig. 6) as well as for stably transfected NF54^[hGrx1–roGFP2]^-*attB* parasites (Fig. 7). The disturbance was time and dose dependent and already occurred at very low micromolar ML304 concentrations. Recently, it was shown that the dynamics of hydrogen peroxide are also affected by ML304. Employing stable integration of the H_2_O_2_ redox probe roGFP2-Orp1 into blood stages of NF54-*attB*, ML304 was found to significantly increase the cytosolic H_2_O_2_ levels [48]. It can, therefore, be deduced that inhibition of the G6PD reaction, likely followed by impairment of NADPH fluxes, markedly affects antioxidant capacity in *Plasmodium* parasites. This is further supported by the finding that ML304 and the redox cycler methylene blue have synergistic effects on late stage gametocytes [56]. The redox-cycling activity of methylene blue leads to consumption of NADPH, while the simultaneous inhibition of *Pf*GluPho by ML304 prevents the generation of new NADPH. Currently, this concept is being followed up.

#### Discovery of novel G6PD inhibitors

Based on a chemical compound screening and following SAR studies, a set of new potential G6PD inhibitors was synthesized and assessed (Fig. 8). In fact, this approach resulted in two new compounds with nanomolar activity against *Pf*GluPho and/or *Pv*G6PD, which will be followed up in detail.

In the past, it was demonstrated that nitro-activated perchloroalkenes are unique building blocks for the synthesis of many microbiologically active, highly substituted heterocycles. Most of the tested compounds have been synthesized this way. One of the big advantages to this approach is the feasibility of modifying and tailoring the resulting heterocycles [69–71].

## Conclusion

The glucose 6-phosphate dehydrogenase 6-phosphogluconolactonases of *P. falciparum* and *P.* *vivax* share many features. Both are bifunctional enzymes catalyzing the first two steps of the oxidative PPP, thereby producing 6-phosphogluconate and NADPH. They share high amino acid identity, conserved substrate binding sites, and kinetic properties. All compounds tested in this study inhibit *Pf*GluPho and *Pv*G6PD with comparable efficacy and modes of action. With ellagic acid, ML304, and compound **4** (vz1732), promising inhibitors with micro- to nanomolar activity are now on hand. It has furthermore been demonstrated that ML304 markedly disturbs the glutathione redox balance of *Plasmodium* parasites, thus further proving its mechanism of action. We therefore propose to following up the development of G6PD inhibitors as novel anti-malarial agents, bearing in mind that it might be possible to develop one compound that is equally active against both *P. falciparum* and *P. vivax*.

## Additional files

**Additional file 1.** Multi-amino acid sequence alignment of G6PDs from different species. The C-terminally located Ser–Ser pair (S899 and S900) found to be phosphorylated in *P. falciparum* is specific for malaria parasites. *Pf*, *Plasmodium falciparum* (PlasmoDB PF3D7_1453800); *Pv, Plasmodium vivax*, (PlasmoDB PVX_117790); h, human (NCBI CAA39089.1); *At*, *Arabidopsis thaliana* (NCBI BAE99888); *Sc*, *Saccharomyces cerevisiae* (NCBI CAA93357). | Identical residues, : very similar residues, . similar residues.

**Additional file 2.** Oligonucleotide primers used for site-directed mutagenesis of *P. falciparum* GluPho.

**Additional file 3.** Synthesis of G6PD inhibitors.

**Additional file 4.** Substrate concentrations for the kinetic characterization of *Pf*GluPho, *Pv*G6PD, and hG6PD.

**Additional file 5.** Amino acid sequence alignment of *Pf*GluPho (PF3D7_1453800) and *Pv*GluPho (PVX_117790). | Identical residues, : very similar residues, . similar residues.

## Abbreviations

6PGL: 6-phosphogluconolactonase
ATC: artemisinin-based combination therapy
ATS: artemisinin
CEA: coruleoellagic acid
CLSM: confocal laser scanning microscopy
CQ: chloroquine
DIA: diamide
EA: ellagic acid
EC_50_: effective concentration
FEA: flavellagic acid
G6P: glucose 6-phosphate
G6PD: glucose 6-phosphate dehydrogenase
GluPho: glucose 6-phosphate dehydrogenase 6-phosphogluconolactonase
hGrx1–roGFP2: human glutaredoxin 1-reduction–oxidation sensitive green fluorescent protein 2
IC_50_: inhibitory concentration
IPTG: isopropyl-β-d-thiogalactopyranoside
NADP^+^: nicotinamide adenine dinucleotide phosphate
NEM: *N*-ethylmaleimide
wt: wild type
