# Supplementary material for: Glucose 6-phosphate dehydrogenase 6-phosphogluconolactonase: characterization of the Plasmodium vivax enzyme and inhibitor studies
Source: Malar J. 2019 Jan 25;18:22. doi: 10.1186/s12936-019-2651-z (PMC6346587; doi:10.1186/s12936-019-2651-z)
Supplement: Supplementary file 1 — Additional file 1. Multi-amino acid sequence alignment of G6PDs from different species. The C-terminally located Ser–Ser pair (S899 and S900) found to be phosphorylated in P. falciparum is specific for malaria parasites. Pf, Plasmodium falciparum (PlasmoDB PF3D7_1453800); Pv, Plasmodium vivax, (PlasmoDB PVX_117790); h, human (NCBI CAA39089.1); At, Arabidopsis thaliana (NCBI BAE99888); Sc, Saccharomyces cerevisiae (NCBI CAA93357). | Identical residues, : very similar residues, . similar residues. [file 12936_2019_2651_MOESM1_ESM.docx]

**Additional file 1. Multi-amino acid sequence alignment of G6PDs from different species.**

**899/900**

*Pf*G6PD LKELQEK-QVKPLKYSFGSSGPKEVFGLVKKYY-NY--GKNYTHRPEFVRK**SS**FYEDDLL 907

*Pv*G6PD LNELQEK-KVQPLKYPFGSSGPKEVYDLVKKYY-NY--GKNYANTPAFVRK**SS**FYEDDLL 924

*Sc*G6PD LKHIERPDGPTPEIYPYGSRGPKGLKEYMQKHKYVMPEKHPYAWPVTKP----EDTKDN- 505

hG6PD LHQIELE-KPKPIPYIYGSRGPTEADELMKRVGFQY--EGTYKWVNPHK----L------ 515

*At*G6PD LHRIDKG-EVKSIPYKPGSRGPKEADQLLEKAGYLQ--THGYIWIPPTL----------- 515

|:.:: | || ||. ::: |

*Pf*G6PD DIN 910

*Pv*G6PD DIN 927

*Sc*G6PD --- 505

hG6PD --- 515

*At*G6PD --- 515
