# Supplementary material for: Glucose 6-phosphate dehydrogenase 6-phosphogluconolactonase: characterization of the Plasmodium vivax enzyme and inhibitor studies
Source: Malar J. 2019 Jan 25;18:22. doi: 10.1186/s12936-019-2651-z (PMC6346587; doi:10.1186/s12936-019-2651-z)
Supplement: Supplementary file 2 — Additional file 2. Oligonucleotide primers used for site-directed mutagenesis of P. falciparum GluPho. [file 12936_2019_2651_MOESM2_ESM.docx]

**Additional file 2. Oligonucleotide primers used for site-directed mutagenesis of *P. falciparum* GluPho**

| Mutant | Sequence^a^ | |
| --- | --- | --- |
| *Pf*GluPho^S899E^ | Sense | 5'-CTGAGTTTGTTAGAAAAGAGTCTTTTTATGAAGACGATT-3' |
|  | Antisense | 5'-AATCGTCTTCATAAAAAGACTCTTTTCTAACAAACTCAG-3' |
| *Pf*GluPho^S900E^ | Sense | 5'-CTGAGTTTGTTAGAAAATCCGAATTTTATGAAGACGATT-3' |
|  | Antisense | 5'-AATCGTCTTCATAAAATTCGGATTTTCTAACAAACTCAG-3' |
| *Pf*GluPho^S315Y^ | Sense | 5'-AAAATTATACAAAATATATTGAAGAAATTTATG-3' |
|  | Antisense | 5'-CATAAATTTCTTCAATATATTTTGTATAATTTT-3' |
| *Pf*GluPho^L395F^ | Sense | 5'-ATAGTTATATATTTCAAACGATGTTTATTATG-3' |
|  | Antisense | 5'-CATAATAAACATCGTTTGAAATATATAACTAT-3' |
| *Pf*GluPho^F507L^ | Sense | 5'-TCATCAAATTATAATTTGCCATATGTTATAAA-3' |
|  | Antisense | 5'-TTTATAACATATGGCAAATTATAATTTGATGA-3' |

^a^Codons with mutated base pairs are underlined.
