# Supplementary material for: Glucose 6-phosphate dehydrogenase 6-phosphogluconolactonase: characterization of the Plasmodium vivax enzyme and inhibitor studies
Source: Malar J. 2019 Jan 25;18:22. doi: 10.1186/s12936-019-2651-z (PMC6346587; doi:10.1186/s12936-019-2651-z)
Supplement: Supplementary file 3 — Additional file 3. Synthesis of G6PD inhibitors. [file 12936_2019_2651_MOESM3_ESM.docx]

**Additional file 3. Synthesis of G6PD inhibitors.**

**Experimental Section**

The synthetic sequences to the compounds **2 (**vz1731) and **4** (vz1732) are shown in scheme 1, for the compounds **8** (vz0882), **10** (vz1204), and **13** (vz0288) in scheme 2, and for the imidazolidine **18** (vz0909) in scheme 3.

Scheme 1

Scheme 2

Scheme 3

**General information.** Melting points were determined with a Büchi apparatus 520 and are uncorrected. Thin layer chromatography (TLC) was performed on Merck TLC-plates (aluminum based) silica gel 60 F 254. Separation of compounds and purifications were carried out by means of column chromatography on silica gel 60 (Merck). Petroleum ether as eluent had the boiling range 60 – 70 °C. FT-IR spectra were obtained in the range of 400 to 4000 cm*^-^*^1^ with a Bruker Vector 22 FT-IR spectrometer equipped with ALPHA's Platinum ATR single reflection diamond ATR module. Mass spectra were obtained on a Varian 320 MS Triple Quad GC/MS/MS instrument with a Varian 450-GC unit usually in direct mode with electron impact (70 eV). In the case of chlorinated and brominated compounds, all peak values of molecular ions as well as fragments refer to the isotopes ^35^Cl and ^79^Br. The elemental composition was confirmed by high-resolution EI and (+)-ESI mass spectrometry. All HRMS results were satisfactory in comparison to the calculated accurate masses of the molecular ions (*±*2 ppm, R *∼* 10000). ^1^H NMR (600 MHz), ^13^C NMR (150 MHz): Avance III 600 MHz FT-NMR spectrometer (Bruker, Rheinstetten, Germany); ^1^H NMR (400 MHz), ^13^C NMR (100 MHz): Avance 400 FT-NMR spectrometer (also Bruker). ^1^H NMR (200 MHz), ^13^C NMR (50 MHz): DPX 200 FT-NMR spectrometer (also Bruker). ^1^H and ^13^C NMR spectra were referenced to the residual solvent peak: CDCl_3_: δ = 7.26 (^1^H), δ = 77.0 (^13^C) ppm; DMSO-*d*_6_: δ = 2.50 (^1^H), δ = 39.7 (^13^C) ppm. Chemical shifts δ are given in ppm.

**Starting Material**. Nitrotrichloroethene (**6**) was synthesized according to the literature [4] by nitration of trichloroethene (**5**) with a 10:1 solution of 63% HNO_3_ and 98% H_2_SO_4_ in 31% yield (bp 56–57 °C/25 mbar). 1,1-Bis(benzotriazol-1-yl)-2-nitro-2-chloroethene (**7**) was prepared according to the literature [5] from the nitroethene **6** and 1*H*-benzotriazole. Yield: 83%. Dimerization of ethene **5** with benzoyl peroxide [6] and subsequent dehydrochlorination of dimer (1,1,3,3,4,4-hexachlorobut-1-ene) in the presence of a catalytic amount of chlorosulfonic acid at 95-100 °C or with dry iron trichloride at 125-1​30 °C gave butadiene **9** [7]. 2-Nitropenta-chlorobuta-1,3-diene (**11**) was synthesized according to the literature [8] from diene **9** with a 10:1 solution of 63% HNO_3_ and 98% H_2_SO_4_ in 53% yield (bp 69–71 °C, 1 mbar). Trichloroacroleine (**12**) was found as a sideproduct (8%) and separated by distillation (bp 57-58 °C at 12 mbar). All spectral data were in accordance with the literature. All other chemicals such as pyrimidine **1**, pyridines **3** and **15**, and diamine **14**, used in this study were commercially available.

**Syntheses**

**2-Bromo-2-(pyrimidin-2-yl)acetonitrile (2**, vz1731**)**. To a solution of pyrimidine **1** (1.20 g, 10.00 mmol) in 25 mL water at 0-5 °C was added dropwise in 10 min bromine (1.67 g, 0.54 mL, 10.50 mmol) and the resulting mixture was stirred for 4 h at the same temperature . Then cold water (50 mL) was added under vigorous stirring. After extraction with chloroform (3 *×* 40 mL) the combined organic layers were washed with water (2 x 50 mL) and dried (calcium chloride). The crude product was purified by means of column chromatography (petroleum ether - ethyl acetate 2 : 1 as eluent). Evaporation of solvents gave 1.11 g **2** (vz1731) as a brown solid. Yield 56%; m. p. 68 – 70 °C. – IR: ν = 3081, 2934, 1566, 1415, 999, 822, 663, 633, 458 cm^−1^. – ^1^H NMR (400 MHz, CDCl_3_): δ = 8.84 d (2H, *J* = 4.9 Hz, CH), 7.35 t (1H, *J* = 4.9 Hz, CHr), 5.65 s (1H, CHBr). – ^13^C NMR (100 MHz, CDCl_3_): δ = 162.4 (Cq), 158.4 (2 CH), 121.1 (1 CH), 114.8 (CN), 29.3 (CH). MS (EI, 70 eV): *m/z* (%) = 197 (17) [M^+^], 118 (100) [M - Br]^+^, 91 (21) [M - Br - HCN]^+^. HRMS-EI: calcd. for C_6_H_4_BrN_3_ [M]^+^: 196.9589; found: 196.9690.

**2-Bromo-2-(3-chloropyridin-2-yl)acetonitrile (4**, vz1732**)**. To a solution of pyridine **3** (158 mg, 1.00 mmol) and NBS (187 mg, 1.05 mmol) in abs. 1,2-dichloroethane (10 mL) AIBN (16.4 mg, 0.1 mmol) was added in portions within 1 h under N_2_ atmosphere and reflux. Subsequently, the supernatant liquid was concentrated *in* *vacuo*, diluted with ice water (20 mL) and extracted with DCM (3 x 15 mL). The combined organic layers were washed with water (1 x 20 mL) and dried (calcium chloride). The crude product was purified by means of column chromatography (petroleum ether - ethyl acetate 2 : 1 as eluent). Evaporation of the solvents gave 153 mg **4** (vz1732) as a yellowish solid. Yield 66%; m. p. 52 – 54 °C. – IR: ν = 3057, 2995, 1568, 1428, 1416, 1136, 1035, 814, 669, 619 cm^−1^. – ^1^H NMR (400 MHz, CDCl_3_): δ = 8.62 dd (1H, *J* = 4.6, 1.5 Hz, CH), 7.78 dd (1H, *J* = 8.2, 1.5 Hz), 7.36 dd (1H, *J* = 8.2, 4.6 Hz, CH), 5.93 s (1H, CHBr). – ^13^C NMR (100 MHz, CDCl_3_): δ = 149.1 (Cq), 148.3 (CH), 138.4 (CH), 130.3 (CCl), 126.1 (CH), 114.8 (CN), 26.2 (CH). MS (EI, 70 eV): *m/z* (%) = 230 (10) [M^+^], 151 (100) [M - Br]^+^, 124 (22) [M - Br - HCN]^+^, 116 (21) [M - Br - Cl]^+^. HRMS-ESI: calcd. for C_7_H_5_BrClN_2_ [M+H]^+^: 230.9325; found: 230.9327.

**1,1- Bis(benzotriazol-1-yl)-2-chloroethene (8**, vz0882 **)**. To a suspension of diene **7** (342 mg, 1.00 mmol) in methanol (20 mL) at r.t. potassium thiocyanate (195 mg, 2.00 mmol) was added and the resulting mixture was stirred at r.t. for 6 h and at 25-30 °C for 2 d. Subsequently, the supernatant liquid was concentrated *in* *vacuo* to a volume of about 5 mL, diluted with cold water (40 mL) and conc. HCl (1 mL). After stirring for 10 min, the precipitate was filtered off, washed with water (2 × 10 mL) and cold MeOH (2 mL) and finally dried under reduced pressure to yield 119 mg (40%) of **8** (vz0882) as a yellowish solid; m. p. 129 – 130 °C. – IR: ν = 2964, 1625, 1603, 1488, 1450, 1375, 1179, 1076, 959, 789, 744, 617 cm^−1^. – ^1^H NMR (200 MHz, CDCl_3_): δ = 8.41 s (1H, *J*^13^_C-H_ = 186 Hz, CHCl), 8.06-8.23 m (2H, CH), 7.32-7.56 m (4H, CH), 7.17-7.27 m (1H, CH), 7.04-7.12 m (1H, CH). – ^13^C NMR (50 MHz, CDCl_3_): δ = 146.8 (Cq), 146.3 (Cq), 132.5 (Cq), 131.5 (Cq), 130.1 (CH), 130.0 (CH), 125.9 (CH), 125.5 (CH), 121.0 (CH), 120.9 (CH), 110.2 (CH), 110.1 (CH), 93.9 (CHCl), 84.7 (Cq). MS (EI, 70 eV): *m/z* (%) = 296 (25) [M^+^], 268 (18) [M - N_2_]^+^, 233 (30) [M - N_2_ - Cl]^+^, 205 (78), 178 (22) [M - benzotriazolyl]^+^. HRMS-ESI: calcd. for C_14_H _9_ClN_6_Na [M+Na]^+^: 319.0475; found: 319.0479.

**2,4,4-trichloro-2-nitrobut-3-enoic acid (10**, vz1204**)** was synthetized in accordance to the literature [9] from 2.26 g (10.0 mmol) **9** and fuming nitric acid (20 mL) at 0 °C. Yield 1.73 g (74%). White solid; m. p. 91– 93 °C. – IR: ν = 3424, 3082, 2604, 1715 (CO), 1639, 1598 (NO_2_), 1404, 1317 (NO_2_), 1225, 1106, 897, 810, 677, 602 cm^−1^. – ^1^H NMR (200 MHz, CDCl_3_): δ = 11.43 s (1H, OH), 7.24 s (1H, *J*^13^_C-H_ = 168.6 Hz, CH). – ^13^C NMR (50 MHz, CDCl_3_): δ = 167.3 (CO), 141.2 (Cq), 123.0 (CH), 110.0 (Cq). MS (EI, 70 eV): *m/z* (%) = 233 (3) [M^+^], 187 (90) [M - NO_2_]^+^, 170 (12) [M - NO_2_ - OH]^+^, 159 (63), 151 (32) [M - NO_2_ - HCl]^+^.

In the literature [9] this compound was identified as 3,4,4-trichloro-2-nitrobut-3-enoic acid. However, this structure proposal is false. The true structure of this molecule is 2,4,4-trichloro-2-nitrobut-3-enoic acid. It was verify by INADEQUATE spectra of this compound through ^13^C-^13^C-constants. The shift of the CH-atom in vz1204 located by 123 ppm, which is typical for CCl_2_ = CH group. The signal of the carbon atom of the C-NO_2_ group is wide, located by 110 ppm and keeps not H (DEPT shows CH at 123 ppm). According to our results of some polychlorobutadienes and –butenes the ^1^*J* (^13^C-^13^C) coupling constant within CCl_2_ = CH group must be located between 89 and 96 Hz, so we have in vz1204 unequivocal confirmation of CCl_2_ = CH group with ^1^*J* (^13^C-^13^C) = 89.2 Hz (Figure 1).

Figure 1. INADEQUATE and ^13^C-NMR spectas of vz1204

**2-Chloro-3,3-diiodoacrylaldehyde (13**, vz0288**)**. Aldehyde **12** (1.6 g, 10 mmol) was added at 0 °C dropwise within 5 min to 15 mL 58% aqueous hydriodic acid; the resulting mixture was stirred at 0-5 °C for 2 h and at r.t. for 3 h. The precipitate was filtered off, washed with water (3 × 30 mL) and hexane (2 x 5 mL) and finally dried under reduced pressure to yield 119 mg (60%) of **13** (vz0288) as a brown solid; m. p. 49 – 50 °C. – IR: ν = 1661, 1518, 1483, 1373, 1119, 758, 710, 653 cm^−1^. – ^1^H NMR (200 MHz, CDCl_3_): δ = 9.86 s (1H, *J*^13^_C-H_ = 193 Hz, CHO). – ^13^C NMR (50 MHz, CDCl_3_): δ = 181.8 (CHO), 137.7 (CCl), 39.4 (CI). MS (EI, 70 eV): *m/z* (%) = 342 (100) [M^+^], 313 (5) [M - CHO]^+^, 254 (26), 215 (7) [M - I]^+^, 127 (95) [I]. HRMS-ESI: calcd. for C_3_H_2_ClI_2_O [M+H]^+^: 342.7884; found: 342.7890.

***N^1^*,*N^2^*-Bis((6-chloropyridin-3-yl)methyl)ethane-1,2-diamine (16)**. A solution of 0.30 g (5.00 mmol) ethane-1,2-diamine in toluene (5 mL) was added dropwise at r.t. to a solution of 1.62 g (10.0 mmol) 2-chloro-5-(chloromethyl)pyridine in 50 mL of the same solvent within 10 min. After stirring for 1 d at r.t., 1.38 g (10.0 mmol) potassium carbonate was added and the mixture was stirred for an additional 3 d. Subsequently, the solvent was removed and water (100 mL) was added to the resulting residue. Extraction with chloroform (3 x 70 mL), washing of the organic phase with water, drying (calcium chloride) and evaporation of the solvents afforded 1.87 g (60%) of **16** as highly viscous brown oil. **16** was also obtained as byproduct of the synthesis of 2-chloro-5-((2-aminoethyl)-aminomethyl)pyridine. [10] ^l^H NMR (200 MHz, CDCl_3_) δ 8.32 (dd, *J* = 0.8, 2.5 Hz, 2H, CH), 7.67 (dd, *J* = 2.5, 8.2 Hz, 2H, CH), 7.28 (dd, *J* = 0.8, 8.2 Hz, 2H, CH), 3.78 (s, 4H, CH_2_), 2.74 (s, 4H, CH_2_), 1.51 (br s, 2H, NH). ^l3^C NMR (50 MHz, CDCl_3_) 149.8 (NCCl), 149.1 (N=CH), 138.5 (CH), 134.6 (Cq), 123.8 (CH), 50.2 (CH_2_), 48.5 (CH_2_).

**1,3-Bis-(2-chloro-5-pyridylmethyl)-2-(2,3,3-trichloro-1-nitro-allylidene)imidazolidine (17)**. To a solution of 934 mg (3.00 mmol) diamine **16** in MeOH (20 mL) a solution of 407 mg (1.50 mmol) nitrobutadiene **11** in MeOH (5 mL) was added at 5 °C within 10 min. The resulting mixture was kept at 5 °C for 2 h, then 3 h at r.t. The precipitate was filtered off, washed with H_2_O (3 × 10 mL), MeOH (1 × 3 mL), and Et_2_O (1 × 5 mL). Drying in vacuo yielded 612 mg (80%) of **17** as a white solid, mp 185-186 °C. ^l^H NMR (200 MHz, DMSO-d_6_) δ 8.39 (d, *J* = 2.4 Hz, 2H, CH), 7.83 (dd, *J* = 2.4, 8.3 Hz, 2H, CH), 7.57 (d, *J* = 8.2 Hz, 2H, CH), 4.54 (s, 4H, CH_2_), 3.87 (s, 4H, CH_2_). ^l3^C NMR (50 MHz, DMSO-d_6_) 163.2 (NCN), 150.0 (NCCl), 149.0 (N=CH), 139.1 (CH), 130.0 (Cq), 125.9 (Cq), 124.5 (CH), 121.5 (Cq), 98.1 (CNO_2_), 49.5 (CH_2_), 48.1 (CH_2_). – IR: ν = 1556, 1533, 1439, 1354, 1324, 1137, 1104, 999, 818, 784, 717, 640 cm^−1^. MS (EI, 70 eV): *m/z* (%) = 507 (2) [M^+^], 472 (4) [M - Cl]^+^, 381 (26) [M - (chloropyridylmethyl)]^+^, 362 (16), 126 (100) [chloropyridylmethyl]^+^. HRMS-ESI: calcd. for C_18_H_15_N_5_Cl_5_O_2_ [M+H]^+^: 507.9668; found: 507.9662.

**3-(1,3-Bis((6-chloropyridin-3-yl)methyl)imidazolidin-2-ylidene)-1,1-dichloro-1,3-dinitro-propan-2-one (18**, vz0909**)**. At 0 °C 510 mg (1.00 mmol) imidazolidine **17** were added to 65% nitric acid (10 mL) within 5 min. The reaction mixture then was stirred for 1 h at 0 °C and an additional 4 h at 10 °C. After pouring onto ice-water (100 mL), the resulting precipitate was filtered off and washed with water (3 x 20 mL). Drying *in vacuo* yielded 402 mg (75%) of dinitropropanone **18** (vz0909) as a white solid, mp 177–179 °C. ^l^H NMR (200 MHz, DMSO-d_6_) δ 8.33 (d, *J* = 1.8 Hz, 1H, NCH), 7.74 (dd, *J* = 1.8, 8.2 Hz, 1H, CH), 7.59 (d, *J* = 8.2 Hz, 1H, CH), 4.46 (s, 4H, CH_2_), 3.96 (s, 4H, CH_2_). ^l3^C NMR (50 MHz, DMSO-d_6_) δ 167.7 (CO), 160.9 (NCN), 150.5 (NCCl), 149.9 (CH), 139.9 (CH), 129.1 (Cq), 124.7 (CH), 109.0 (CNO_2_), 103.7 (CCl_2_NO_2_), 47.4 (CH_2_), 47.1 (CH_2_). IR: 2918, 1610, 1585, 1565, 1459, 1390, 1331, 1139, 1107, 1025, 898, 803, 769, 650, 550, 493. MS (EI, 70 eV): *m/z* (%) = 534 (2) [M^+^], 517 (2) [M - OH]^+^, 442 (5), 336 (15), 126 (100) [chloropyridylmethyl]^+^. HRMS (ESI): *m/z* [M + Na]^+^ calcd for C_18_H_14_N_6_Cl_4_N_6_O_5_Na: 556.9677; found: 566.9673.

**Acknowledgments**

The authors thank Dr. G. Dräger, Leibniz University of Hannover, for HRMS measurements.

SAR (PASS) for imidazolidine **19** (vz0914)

*PASS (Prediction of Activity Spectra for Substances)* is a software product designed as a tool for evaluating the general biological potential of an organic drug-like molecule. *PASS* provides simultaneous predictions of many types of biological activity based on the structure of organic compounds. Thus, *PASS* can be used to estimate the biological activity profiles for virtual molecules, prior to their chemical synthesis and biological testing.

*Pa (probability "to be active")* estimates the chance that the studied compound is belonging to the sub-class of active compounds (resembles the structures of molecules, which are the most typical in a sub-set of "actives" in PASS training set).

*Pi (probability "to be inactive")* estimates the chance that the studied compound is belonging to the sub-class of inactive compounds (resembles the structures of molecules, which are the most typical in a sub-set of "inactives" in PASS training set).


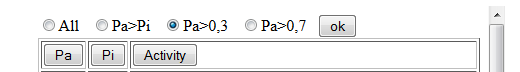


| 0,602 | 0,003 | Insecticide |
| --- | --- | --- |
| 0,563 | 0,001 | Integrin alphaLbeta2 antagonist |
| 0,508 | 0,116 | Nicotinic alpha6beta3beta4alpha5 receptor antagonist |
| 0,432 | 0,014 | Glycogen synthase stimulant |
| 0,412 | 0,041 | Antipsoriatic |
| 0,405 | 0,028 | CF transmembrane conductance regulator agonist |
| 0,387 | 0,127 | Nicotinic alpha2beta2 receptor antagonist |
| 0,358 | 0,167 | Acetylcholine neuromuscular blocking agent |
| 0,308 | 0,003 | Nicotinic alpha3beta4 receptor agonist |
| 0,304 | 0,183 | APOA1 expression enhancer |

|  |
| --- |

|  |
| --- |

**References**

1. Zapol’skii VA, Fischer R, Namyslo JC, Kaufmann DE. Chemistry of polyhalogenated nitrobutadienes, 8: Nitropolychlorobutadienes - Precursors for insecticidal neonicotinoids. Bioorg Med Chem. 2009;17:4206-15.
2. Zapol’skii VA, Namyslo JC, Gjikaj M, Kaufmann DE. Chemistry of polyhalogenated nitrobutadienes, 4: reactions of mono-, bis-, and tris(4-tolylthio) derivatives of 2 nitroperchloro-1,3-butadiene with α,β-bifunctional nucleophiles. ARKIVOC Volume 2007. Part. (i): General Papers. 76-93.
3. Zapol’skii VA, Namyslo JC, Adam AEW, Kaufmann DE. Chemistry of Polyhalogenated Nitrobutadienes, 1: A New Synthesis of Perfunctionalized 3-Amino-4-nitrothiophenes. Heterocycles. 2004;63:1281-98.
4. Meyer C, Zapol’skii VA, Adam AEW, Kaufmann DE. Chemistry of Halonitroethenes, 1: First Synthesis of Functionalized 3-Chloroquinoxalin-2(1H)-one 4-Oxides. Synthesis. 2008;16:2575-81.
5. Zapol’skii VA, Yang X, Namyslo JC, Gjikaj M, Kaufmann DE. Chemistry of Halonitroethenes, III. Part II. Synthesis. 2012;44:885-94.
6. Watanabe T, Ishikaki H, Okata H. Radical dimerization of trichloroethylene. Jpn Kokai Tokkyo Koho. 1998; 10204007
7. Ol’dekop YA, Kaberdin RV, Buslovskaya EE. Studies on acyl peroxides. XLVII. Reaction of acetyl peroxide with trichloroethylene. J Org Chem USSR (Engl. Transl.). 1981;17:222-5.
8. Potkin VI, Zapol’skii VA, Kaberdin RV. Nitration of 2-H-pentachloro-1,3-butadiene. Doklady of the National Academy of Sciences of Belarus. 1996;40:68-71.
9. Ol‘dekop YA, Kaberdin RV. Reaction of 2H-pentachloro-1,3-butadiene with fuming nitric acid. J Org Chem USSR (Engl. Transl.). 1975;11:288-90.
10. Nakamura J, Isogai A. Jpn Kokai Tokkyo Koho. 1995;JP 07242633 A 19950919.
