# Supplementary material for: Glucose 6-phosphate dehydrogenase 6-phosphogluconolactonase: characterization of the Plasmodium vivax enzyme and inhibitor studies
Source: Malar J. 2019 Jan 25;18:22. doi: 10.1186/s12936-019-2651-z (PMC6346587; doi:10.1186/s12936-019-2651-z)
Supplement: Supplementary file 4 — Additional file 4. Substrate concentrations for the kinetic characterization of PfGluPho, PvG6PD, and hG6PD. [file 12936_2019_2651_MOESM4_ESM.docx]

**Additional file 4. Substrate concentrations for the kinetic characterization of *Pf*GluPho, *Pv*G6PD, and hG6PD.**

|  | *Pf*GluPho | | *Pv*G6PD | | hG6PD | |
| --- | --- | --- | --- | --- | --- | --- |
| Substrate… | NADP^+^ [µM] | G6P [µM] | NADP^+^ [µM] | G6P [µM] | NADP^+^ [µM] | G6P [µM] |
| …in saturation | 200 | 200 | 200 | 800 | N/A | N/A |
| …close to *K*_M_ | 10 | 15 | 15 | 70-85* | 19 | 125 |

* Depending on the individual *K*_M_ of the enzyme batch used. N/A: not applicable
