# Supplementary material for: Glucose 6-phosphate dehydrogenase 6-phosphogluconolactonase: characterization of the Plasmodium vivax enzyme and inhibitor studies
Source: Malar J. 2019 Jan 25;18:22. doi: 10.1186/s12936-019-2651-z (PMC6346587; doi:10.1186/s12936-019-2651-z)
Supplement: Supplementary file 5 — Additional file 5. Amino acid sequence alignment of PfGluPho (PF3D7_1453800) and PvGluPho (PVX_117790). | Identical residues, : very similar residues, . similar residues. [file 12936_2019_2651_MOESM5_ESM.pdf]

Additional file 5. Amino acid sequence alignment of *Pf*GluPho (PF3D7_1453800) and *Pv*GluPho (PVX_117790).

```
PfGluPho       1 MDYENFVKSAEEINNLHNVNYLETKDLNDFNWKAAYYICKEIYDKQQINK     50
                 ||.:...||.|::|:||||.|||.|||.|||.|:|||||.:|.:||...:
PvGluPho       1 MDCQALAKSLEQMNHLHNVKYLEAKDLTDFNQKSAYYICHQIAEKQLSKE     50

PfGluPho      51 DGYVVIGLSGGRTPIDVYKNMCLIKDIKIDKSKLIFFIIDERYKSDDHKF    100
                 .|:||||||||:||||||||:.|:||||||.|||||||||||||.|||||
PvGluPho      51 GGHVVIGLSGGKTPIDVYKNIALVKDIKIDTSKLIFFIIDERYKRDDHKF    100

PfGluPho     101 SNYNNIKFLFHNLNINEKEQLYKPDTTKSIVDCILDYNDKIKIMIEKYKK    150
                 ||||||||||.:|.||||||||:|||:|:||:|:.|||:|||.|::||.|
PvGluPho     101 SNYNNIKFLFESLKINEKEQLYRPDTSKNIVECVRDYNEKIKNMVKKYTK    150

PfGluPho     151 VDIAILGMGSDFHIASLFPNIFYNIYMNNYQNNYIYNEKTLDFINNDQ--    198
                 ||||||||||||||||||||||:|||||||||:|||:|.::...|::.
PvGluPho     151 VDIAILGMGSDFHIASLFPNIFFNIYMNNYQNSYIYDESSIKVANSNDTS    200

PfGluPho     199 DNDNLKYLKEYVYFTTTNQFDVRKRITVSLNLLANASSKIFLLNSKDKLD    248
                 |||||..|||||||||||.|||||||||||:||.|||||||||||.||||
PvGluPho     201 DNDNLDLLKEYVYFTTTNNFDVRKRITVSLDLLGNASSKIFLLNSTDKLD    250

PfGluPho     249 LWKNMLIKSYIEVNYNLYPATYLIDTSCTNENVNINNNNNNNNKNKNNYC    298
                 ||||||:|||::|||.||||.||||:.
PvGluPho     251 LWKNMLLKSYVDVNYCLYPAVYLIDSM-----------------------    277

PfGluPho     299 YSNTTVISCGYENYTKSIEEIYDSKYALSLYSNSLNKEELLTIIIFGCSG    348
                   ||||::|||.||.:.:|:||.|..:||::|.|||:.|.||||:|||||
PvGluPho     278 --NTTVVTCGYTNYPQMLEDIYVSNSSLSVHSPSLNRRECLTIIVFGCSG    325

PfGluPho     349 DLAKKKIYPALFKLFCNNSLPKDLLIIGFARTVQDFDTFFDKIVIYLKRC    398
                 ||||||||||:||||||..|||||||:|||||.|.||:||||||.|||||
PvGluPho     326 DLAKKKIYPAIFKLFCNKRLPKDLLIVGFARTAQSFDSFFDKIVGYLKRC    375

PfGluPho     399 LLCYEDWSISKKKDLLNGFKNRCRYFVGNYSSSESFENFNKYLTTIEEEE    448
                 |..|||.|:::||||||.|||:|||::|:||||||||.||||||.:|.|.
PvGluPho     376 LHSYEDLSLTQKKDLLNCFKNKCRYYIGDYSSSESFERFNKYLTQLEREN    425

PfGluPho     449 ----AKKKYYATCYKMN--------------------------GSDYNIS    468
                     |...:.|.....:                          |.....:
PvGluPho     426 LIGTAPTSWAAAAGNASFANDTDKVEHHPDEAILAKGTGAATPGEAPGGA    475

PfGluPho     469 NNVAEDNISIDDENKTNEYFQ-MCTPKNCPDNVFSSNYNFPYVINRMLYL    517
                 |..|....:....|..:...| ...|.:...:...|..|.|:.|||:|||
PvGluPho     476 NGPATHGEAHGGANGLSTPMQGKAVPTDDTSDEGHSGANHPFAINRILYL    525

PfGluPho     518 ALPPHIFVSTLKNYKKNCLNSKGTDKILLEKPFGNDLDSFKMLSKQILEN    567
                 ||||||||||||||||||.|..||||||||||||.||.:||:||||||||
PvGluPho     526 ALPPHIFVSTLKNYKKNCQNGNGTDKILLEKPFGKDLQTFKVLSKQILEN    575

PfGluPho     568 FNEQQIYRIDHYLGKDMVSGLLKLKFTNTFLLSLMNRHFIKCIKITLKET    617
                 |||:.|||||||||||||||||||||||||||||||||||||||||||||
PvGluPho     576 FNEEHIYRIDHYLGKDMVSGLLKLKFTNTFLLSLMNRHFIKCIKITLKET    625
```

```
PfGluPho     618 KGVYGRGQYFDPYGIIRDVMQNHMLQLLTLITMEDPIDLNDESVKNEKIK    667
                 ||||||||||||||||||||||||||||.|||||||||||||||||||||
PvGluPho     626 KGVYGRGQYFDPYGIIRDVMQNHMLQLLALITMEDPIDLNDESVKNEKIK    675

PfGluPho     668 ILKSIPSIKLEDTIIGQYEKAENFKEDEN-----NDD----ESKKNHSYH    708
                 |||.|.||||::||||||.|:||::|::|     :||    |||:|||||
PvGluPho     676 ILKCIQSIKLDNTIIGQYVKSENYQEEKNLSAPPSDDHPVDESKRNHSYH    725

PfGluPho     709 DDPHIDKNSITPTFCTCILYINSINWYGVPIIFKSGKGLNKDICEIRIQF    758
                 ||||||.|||||||||||||||||||||||||||:||||||||||||:||
PvGluPho     726 DDPHIDSNSITPTFCTCILYINSINWYGVPIIFKAGKGLNKDICEIRLQF    775

PfGluPho     759 HNIMGSSDENMNNNEFVIILQPVEAIYLKMMIKKTGCEEMEEVQLNLTVN    808
                 |||||||||:|.||||||||||||.|||||||||||||||||||||||||
PvGluPho     776 HNIMGSSDESMYNNEFVIILQPVEVIYLKMMIKKTGCEEMEEVQLNLTVN    825

PfGluPho     809 EKNKKINVPEAYETLLLECFKGHKKKFISDEELYESWRIFTPLLKELQEK    858
                 .||||..|||||||||||||:|.|||||||||||||||||||||.|||||
PvGluPho     826 HKNKKNYVPEAYETLLLECFRGFKKKFISDEELYESWRIFTPLLNELQEK    875

PfGluPho     859 QVKPLKYSFGSSGPKEVFGLVKKYYNYGKNYTHRPEFVRKSSFYEDDLLD    908
                   :|:||||.|||||||||:.||||||||||||.:.|.||||||||||||||
PvGluPho     876 KVQPLKYPFGSSGPKEVYDLVKKYYNYGKNYANTPAFVRKSSFYEDDLLD    925

PfGluPho     909 IN    910
                 ||
PvGluPho     926 IN    927
```
